# Key Components of Parenting Education Interventions for Preterm Infant–Parent Dyads Admitted to the NICU: A Systematic Review

**DOI:** 10.3390/children13020280

**Published:** 2026-02-18

**Authors:** Welma Lubbe, Iolanthé Marike Kruger, Kirsten A. Donald

**Affiliations:** 1 Division of Developmental Paediatrics, Department of Paediatrics & Child Health, Red Cross War Memorial Children’s Hospital, Neuroscience Institute, University of Cape Town, Private Bag X3, Rondebosch 7701, South Africa; 2 NuMIQ Research Focus Area, Faculty of Health Sciences, North-West University, Private Bag X6001, Potchefstroom 2520, South Africa; 3 AUTHeR Research Unit, Faculty of Health Sciences, North-West University, Private Bag X6001, Potchefstroom 2520, South Africa

**Keywords:** neurodevelopmental care, preterm infant, parent education interventions, programmes, training

## Abstract

**Highlights:**

**What are the main findings?**

- Parental education interventions in NICU settings consistently centre on three core components: educational content, programme structure, and integrated parental support.
- Key educational content areas included NICU orientation, infant health/behaviour, infant care (particularly feeding), parental well-being and discharge planning.

**What are the implications of the main findings?**

- The lack of standardised programme structures and limited reporting on intervention development justify the need for clearer, evidence-based design and implementation guidance.
- Greater emphasis is needed on context-specific, culturally responsive interventions, particularly in low-resource settings, to ensure equitable access and improved outcomes.

**Abstract:**

Background: Parents of preterm infants face significant emotional, informational, and caregiving challenges during neonatal intensive care unit (NICU) hospitalisation. Educational interventions are increasingly used to support parental readiness; however, considerable variation exists in their content, structure, and delivery. A clearer understanding of these components is essential to inform the development of effective, contextually responsive programmes. Aim: To identify and synthesise the core educational components, programme structures, and embedded parental support needs within NICU-based educational interventions for parents of preterm infants. Methods: A systematic search of peer-reviewed literature (January 2010–September 2022) identified 33 studies of high methodological quality. Data were extracted and synthesised using thematic analysis. Results: Three overarching domains were identified: (1) educational content, (2) programme structure and delivery, and (3) parental support needs integrated within educational delivery. The educational content encompassed the NICU environment, infant health and behaviour, caregiving practices, parental well-being, and discharge preparation. Programme structures varied widely in terms of intensity, duration, delivery modality, and facilitator roles, with limited justification for structural choices. Parental support–emotional, relational, and confidence-building–was inconsistently embedded despite evidence of its importance. Established interventions such as COPE, FICare, and FCC have clearer theoretical foundations and more holistic support than most locally developed programmes. Conclusions: NICU educational interventions positively influence parental knowledge, confidence, and parent–infant interaction; however, substantial variation and limited conceptual grounding hinder their comparability and scalability. The evidence base remains dominated by high-income settings, which limits its global applicability. Future research must prioritise theory-informed design, transparent reporting, and context-sensitive adaptation, particularly in under-resourced health systems, to support equitable and effective parental education for families of preterm infants worldwide.

## 1. Background

Preterm birth affects approximately 1 in 10 infants, with an estimated 13.4 million infants born early in 2020 [1,2,3,4]. Prematurity not only affects infants but also places significant psychological and emotional strain on parents. Parents of infants in the neonatal intensive care unit (NICU) face complex stressors, including an abrupt and unexpected transition to parenthood. They must adapt to a caregiving role within a highly medicalised environment, often under emotionally intense and uncertain circumstances. This includes coping with their newborns’ fragility and illness while feeling unprepared and restricted in their roles as parents. Parental distress can directly affect infant outcomes. Steinhardt et al. [5] found that parental anxiety may disrupt neurological development, weaken bonding, and impair children’s cognitive and social development. The cascade of adverse effects in preterm infants and their parents can lead to dysfunctional parental relationships, which have also been shown to negatively affect infant development [6,7]. Thus, emotional and educational support for parents is critical to enhancing family well-being.

### 1.1. Effect of Preterm Birth on the Infant

The effects of prematurity on infants are both short- and long-term in nature. In the immediate neonatal period, infants face complications, including physiological instability, risk of intraventricular haemorrhage, respiratory distress, cardiac issues such as patent ductus arteriosus, behavioural stress, and interrupted sleep [8]. Long-term challenges range from developmental delays to more specific neurodevelopmental and behavioural problems. Over half of children born prematurely exhibit impairments in attention, socio-emotional development, executive function, and language or motor skills [9,10]. Those born before 32 weeks face higher risks of moderate to severe challenges by school age [10,11].

The likelihood of conditions such as Attention-Deficit/Hyperactivity Disorder (ADHD) (three times more likely [12]), autism spectrum disorder (up to four times more likely [13]), and cerebral palsy (up to 15% prevalence [14]) is significantly increased in preterm populations. Sensory impairments are also more common, with up to 2.4 times for visual issues and a 2.5% prevalence of hearing loss [15]. As many as 60.9% of infants born at 22 weeks of age have moderate-to-severe neurodevelopmental impairment [16]. Long-term effects can include anxiety rooted in early medical trauma [8], children meeting mental health disorder criteria by age 13 [17], and lower academic achievement and intelligence, which are associated with lower socio-economic status in adulthood [18].

### 1.2. Effect of Preterm Birth on the Parents

For parents, the impact of preterm birth extends well beyond the initial hospitalisation. In the short term, many experience stress, trauma, guilt, anxiety, helplessness, and grief over the loss of a ‘normal’ birth experience and experience helplessness and feelings of an inability to care for and protect their infant [19,20]. Parental roles may be restricted by the medicalised environment, contributing to feelings of inadequacy regarding their parenting role [21,22]. If not adequately addressed, these stressors can contribute to longer-term issues such as peripartum depression, post-traumatic stress symptoms [23], attachment difficulties [24,25], lasting impacts on parenting behaviours [26], and challenges within partner relationships.

### 1.3. Parent Education as Intervention Strategy

Parental education has emerged as a critical intervention to support families facing trauma and uncertainty regarding preterm birth and NICU admissions [27]. Structured educational programmes have been shown to reduce parental anxiety, enhance caregiving confidence, and improve parent–infant interactions, all of which positively influence developmental outcomes in preterm children [26,28,29,30]. Steinhardt et al. [5] found that targeted, structured, and actively supportive parental training was more effective than passive involvement in routine care in improving mother–infant interactions. Notably, programmes that teach parents to read and respond to their infants’ cues have been linked to improved white matter development in preterm infants [9,10].

Historical milestones in the field include the introduction of the Newborn Individualized Developmental Care and Assessment Program (NIDCAP) by Als et al. [31], which emphasised individualised, developmentally supportive care involving parents. The Creating Opportunities for Parent Empowerment (COPE) programme, developed by Melnyk [32] in the late 1990s, became one of the most widely studied parental education models. The early 2000s saw a shift toward family-centred care, culminating in the Family Integrated Care (FICare) model, which was introduced in Canada in 2009 [33]. Recently, digital and mobile platforms have expanded the reach and flexibility of educational content [28,34,35].

Parenting educational programmes for preterm infants are typically delivered in three stages: (1) during the NICU stay, (2) in preparation for discharge, and (3) through ongoing support in early childhood, often integrated with early intervention services [36]. These programmes aim to equip parents with the knowledge, skills, and psychological readiness to actively support their infants’ care.

During the NICU stay, structured education has been shown to reduce psychological stress [26,28,29,30], improve parental knowledge of infant caregiving [37], increase caregiving confidence and competence [29,38], and promote maternal adaptation and functioning [39]. Early parent–infant interaction correlates with better language, emotional, and social development [40,41] and understanding infant cues, which supports healthier attachment [25,42]. Sakonidou et al. [43] found that parental involvement and education increase satisfaction with neonatal care. For example, the COPE programme reduced the length of stay in the NICU by four days [44].

After discharge, education continues to play a role in improving patient outcomes. Melnyk et al. [32] and Ferber and Makhoul [45] found that parents reported stronger beliefs about their childcare abilities and improved parent–infant interaction after receiving discharge education. Studies have also demonstrated fewer symptoms of parental depression and anxiety [46,47,48], lower readmission rates [44], and lower infant morbidity in premature infants [49] after discharge when parents receive discharge education prior to discharge.

### 1.4. Definition of Parent Education

Broom et al. [50] outlined four core components of the FICare model: bedside education and care participation, group education, family-centred rounds, and psychosocial support. Similarly, Benzies et al. [51] noted that education is commonly embedded in early interventions, even when it is not the primary focus. The American Academy of Family Physicians [52] defines patient education as “the process of influencing patient behaviour and producing changes in knowledge, attitudes, and skills necessary to maintain or improve health”. In the NICU, education may include informal bedside coaching or structured sessions covering infant development, feeding, and parental self-care topics. It may also be integrated into therapeutic models such as parent-mediated interventions, where parents act as quasi-therapists [53].

Although structured education is a critical component of neonatal care, it is often embedded within broader psychosocial or multi-component interventions and rarely analysed independently. The lack of specificity limits clarity, hinders replication, and makes it difficult to assess its contributions. For example, Benzies et al. [51] reviewed early interventions without isolating the educational components. Addressing this gap, the present review focuses on structured parenting education delivered during NICU hospitalisation.

This is particularly relevant when considering the contextual disparities. Most existing programmes, such as COPE and NIDCAP, were developed in high-resource settings with access to technology, skilled staff, and stable healthcare systems. Orr et al. [34] noted that most Canadian NICU parents accessed health information via smartphones and preferred digital content. However, such delivery models may not be feasible in low-resource settings with limited electricity, data access, or staff-training capacity.

The evolution of NICU care over the past decade has increased parental involvement globally, mainly due to the FICare model and the rise of digital educational tools. However, as Ahlqvist-Björkroth et al. [54] noted, NICU parenting interventions vary widely, and there is an urgent need for consistent definitions and precise descriptions of educational content. Greater standardisation is essential to facilitate comparisons across settings and promote equity in neonatal care.

This review addresses this need by synthesising and comparing hospital-based parenting education interventions that explicitly focus on the NICU hospitalisation period. By identifying the core components and modalities used to educate parents during this critical stage, we aim to inform the development of effective, inclusive, and scalable educational models that support the well-being of preterm infants and their families in the future.

## 2. Methods

A pragmatic approach, similar to the Joanna Briggs Institute (JBI) approach to systematic reviews, was followed to summarise the best available evidence without restricting the review to randomised controlled trials [55]. Nested within a larger study and as phase 1 thereof, this review aimed to identify the key components of parental education interventions for preterm infant-parent dyads in the NICU. The review adhered to a previously published protocol (Systematic Review Registration: PROSPERO CRD42023398817) and was conducted in accordance with the 2020 Preferred Reporting Items for Systematic Reviews and Meta-Analyses (PRISMA) guidelines [56]. The completed PRISMA 2020 checklist is provided in Supplementary Materials.

### 2.1. Procedure

This review builds on the five-step review process described by Lubbe et al. [57], which includes: (1) the review question, (2) the selection (sampling) process, (3) quality assessment of selected studies, (4) data extraction, (5) results: synthesis and thematic analysis, and (6) presentation of data (discussion).

### 2.2. Review Question

The review question was constructed using the population (P), intervention (I), outcomes (O), and time (T)—the PIOT format [58]. The population refers to all published documents that focused on the parents of preterm infants, while the intervention of interest is the components (topics) of parental education interventions. Time refers to the period during which parental education was presented, as reported in the selected documents, from birth to the hospital discharge. The PIOT review question was thus formulated as follows: What are the key components of parental education interventions (I) presented to parents of preterm infants (P) while the infant was admitted to the NICU (T), which could influence the short- and long-term outcomes of the infant and parents (O)?

### 2.3. Selection Process

#### 2.3.1. Inclusion and Exclusion Criteria

Publications were included if they described educational interventions aimed at the parents of preterm infants from birth to hospital discharge.

#### 2.3.2. Date Range

This review included studies published between 2010 and 2022 to capture contemporary neonatal care practices, evolution of family-centred care models, and advancements in the digital delivery of parental education. Although foundational programmes such as the NIDCAP and COPE were developed before 2010, the past decade has seen a marked expansion of structured, scalable, and technology-enabled educational interventions. More recent literature also better reflects implementation realities in both high- and low-resource settings and aligns more closely with current clinical, policy, and equity considerations. To ensure comprehensive coverage of relevant programme components, the reference lists of all included studies were manually searched for earlier publications describing intervention content or educational frameworks introduced prior to 2010, but still relevant.

#### 2.3.3. Study Restrictions

Only interventions delivered entirely or partially during the NICU stay were included to ensure relevance to the NICU. Studies which focused exclusively on discharge preparation or post-discharge home care were excluded, even if delivered while the infant remained in the NICU. However, interventions that combined education relevant to hospital stay with components addressing discharge or transition to home were included, with the analysis focused on hospital-based elements.

This review included all study designs, as the presentation of educational programmes does not favour a single approach. Qualitative studies have described the components of parent education from various perspectives and for various participants. In addition, randomised controlled trials (RCTs), quasi-experimental studies, and observational studies captured both the efficacy and real-world implementation of parenting education interventions. Published protocols describing educational interventions were also included up to the full-text screening stage to assess whether subsequent publications reported outcomes or described the intervention components. Review studies were excluded, as the primary studies cited in those reviews were individually considered for inclusion and further analyses.

Studies that reported on the effectiveness of an intervention were included at the full-text screening level if the title or abstract suggested that the educational content or components would be described in the text. Studies that described only intervention effectiveness but did not include educational components were excluded. In cases where an intervention was referenced but not described in detail, the study’s reference list was manually searched to locate publications outlining the corresponding programme content.

Interventions classified as neonatal therapy, defined by Khurana et al. [59] as interventions delivered directly to the infant or parent by a healthcare professional (e.g., physical, occupational, or speech therapy) or delivered to parents under therapist guidance, were excluded from the study.

Studies focused on special populations, including infants with specific neonatal conditions (e.g., congenital abnormalities, cardiac disease, cerebral palsy, and those requiring surgery) or maternal populations with unique educational needs (e.g., adolescent mothers or mothers with substance use disorders), were excluded because of the highly specialised nature of their educational requirements during the NICU stay.

This review excluded grey literature and unpublished studies to ensure methodological rigor and peer-reviewed quality assurance. Although grey literature can provide valuable insights, the focus was limited to published journal articles to maintain consistency in data quality, intervention details, and comparability across studies. Only peer-reviewed articles indexed in academic databases were included in this review.

Publications without English abstracts and those published in other languages with English abstracts were excluded. Although electronic translation tools are increasingly available, full-text articles published in languages other than English were excluded due to limited resources for verifying translated methodological details and intervention content with sufficient accuracy.

### 2.4. Search Strategy

A health sciences librarian and the first author developed the search strategy. Keywords identified during a preliminary literature review included neurodevelopmental care, premature infants, parent education interventions, programs, and training. Boolean operators (AND, NOT, OR) and the wildcard symbol (*) were used to refine the search. The search string included: parenting education OR parenting program OR parent* training AND preterm OR premature OR preterm AND infant* OR baby* OR newborn OR neonate* AND intervention* AND NICU OR neonatal intensive care unit OR special care OR newborn intensive care OR baby unit. We searched the following databases and platforms: MEDLINE (via PubMed), CINAHL (via EBSCOhost), Scopus, Web of Science, Cochrane Library, and ScienceDirect.

**Figure 1:**
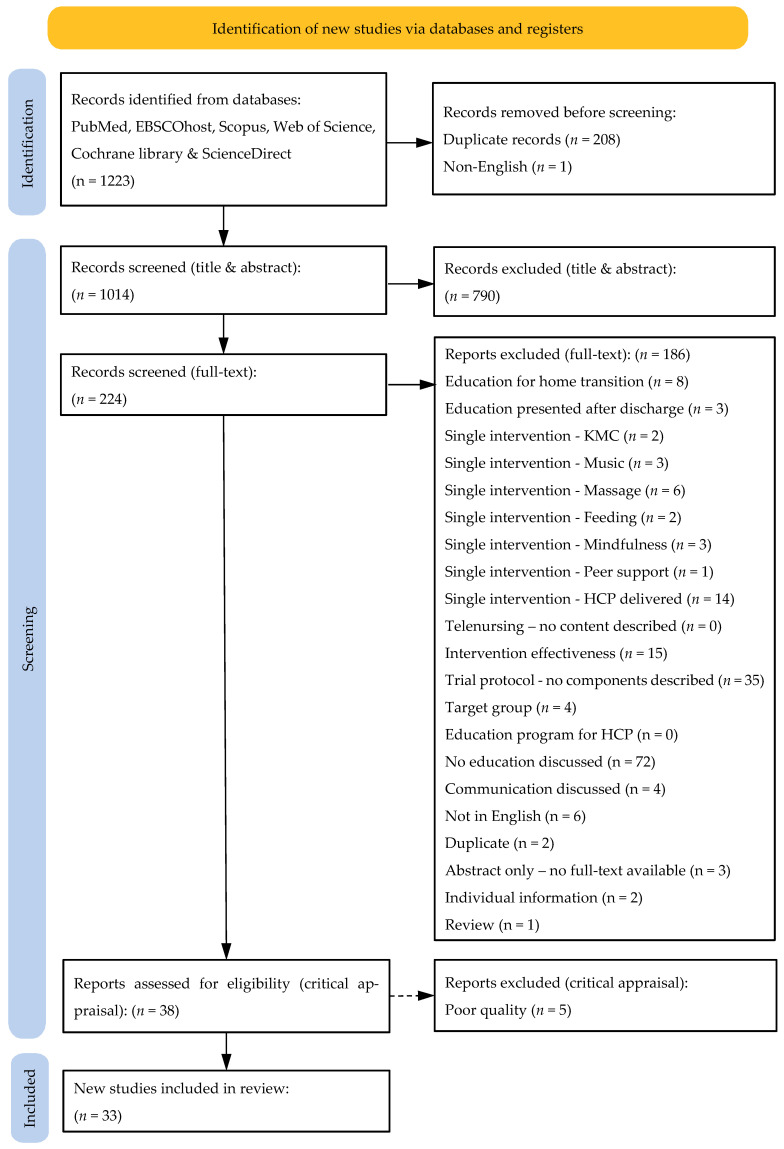
PRISMA diagram: Adapted from Page et al. [56], Page et al. [60], and Haddaway et al. [61].

To ensure a good audit trail and replicability, the process was documented in a flow diagram provided by the PRISMA group [56] (Figure 1).

### 2.5. Title and Abstract Screening

Following database searching, citations were exported to a bibliographic manager, where duplicate records were identified and removed using a built-in duplicate removal function. This process yielded a unique set of records for screening [62]. Title and abstract screening were conducted independently by two reviewers (WL and IMK) to identify articles that did not meet the predefined inclusion criteria. The screening process was recorded using the Evidence for Policy and Practice Information (EPPI) Reviewer software (version 4.12.0.0) [63]. A comparison report was generated, and consensus discussions were held to resolve discrepancies.

During the title and abstract screening, initial disagreements occurred between the reviewers. These were resolved through discussion and refinement of the operational definition of parental education, as described in the Section 1 and Section 2.3 of the manuscript. Records were excluded either for the same or similar reasons by both reviewers or for differing reasons, where multiple exclusion criteria were applied to a single record. Consensus was reached for all exclusions prior to progression to full-text screening.

Although assessing intervention effectiveness was not the primary aim of this review, it was anticipated that studies evaluating intervention outcomes would also describe educational components. A conservative approach was adopted during screening to minimise the risk of excluding relevant material. If a parental education programme was mentioned in the abstract without sufficient detail, the study was retained for full-text review under the assumption that relevant educational content might be reported in the full text. This approach supported the comprehensive identification of educational components, even when these were not the primary focus of the study or were described across multiple publications.

A summary of the number of records screened, excluded, and retained following title and abstract screening is provided in the Section 3 and is illustrated in the PRISMA flow diagram (Figure 1).

### 2.6. Full-Text Screening

The next phase involved retrieving and reviewing the full texts of records identified as potentially eligible following title and abstract screening. A total of 224 full-text articles were retrieved and independently double-reviewed by two reviewers, with discrepancies resolved through comparison of reports and consensus discussions. Studies were assessed against predefined inclusion and exclusion criteria to determine eligibility for synthesis.

Studies assessing the effectiveness of interventions without describing their educational components were excluded. However, when an intervention name was provided, it was documented for potential targeted follow-up. During the full-text screening, the studies were further assessed for the inclusion of educational elements. Where educational components were insufficiently described in the included articles, additional publications related to the same intervention were searched to identify relevant content; original study authors were not contacted for this purpose.

Parent-mediated interventions, including those focused exclusively on feeding, massage, music therapy, mindfulness, kangaroo mother care, communication, or peer support, were excluded, as they lacked explicit descriptions of global educational components and instead emphasised therapeutic or relational aspects (see Section 1).

In addition, full texts were unavailable for three records, and several records were excluded due to language limitations or duplication. Following full-text screening, 38 articles were retained for critical appraisal. A detailed account of the number and reasons for article exclusion during the full-text screening phase is provided in the PRISMA flow diagram (Figure 1).

### 2.7. Quality Assessment of Selected Studies

To evaluate the methodological quality and rigor of the included studies, three validated critical appraisal tools were used based on study design: the JBI checklists [64], the Johns Hopkins Evidence-Based Practice Research Appraisal Tool [65], and the Mixed Methods Appraisal Tool (MMAT, version 2018) [66,67]. These tools assess aspects such as study design appropriateness, methodological rigor, results analysis, and the application of ethical principles.

Two independent reviewers evaluated the quality of the studies. Discrepancies were resolved through discussions and consensus. Studies were included in the review if they achieved a quality rating greater than 50% based on the scoring criteria of the respective appraisal tool. This threshold was applied consistently across all study types, despite the use of different appraisal tools, to ensure the comparability of methodological quality. In total, 33 studies met the quality threshold and were included in the data extraction. These studies provide sufficient detail on the components of educational interventions for parents of preterm infants in the NICU.

### 2.8. Data Extraction

Data extracted from each study included the authors, article title, setting/country, research question, aims, objectives, methodology, data collection and analysis methods, sample, inclusion and exclusion criteria, tools and interventions described, results, effectiveness, programme name, study limitations, recommendations, and specific educational components (specific topics). Secondary data extracted that provided context for the included studies encompassed the method of delivery, characteristics, presentation of the educational intervention, and key individuals involved in the educational programme.

## 3. Results and Discussion

### 3.1. Data Analysis and Synthesis

Following duplicate removal, 1014 records were screened at the title and abstract levels. Of these, 790 records were excluded based on predefined inclusion criteria. The remaining 224 records were assessed at the full-text level. Following full-text screening, 186 articles were excluded for predefined reasons, which are summarised in the PRISMA flow diagram (Figure 1). Thirty-eight articles were retained for critical appraisal, of which 33 met the quality threshold and were included in the final synthesis.

**Figure 2:**
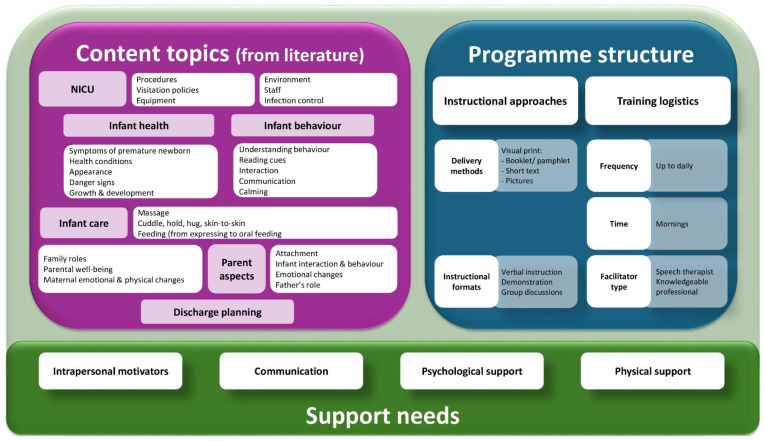
Components of an education intervention for parents of preterm infants in the NICU.

Each of the studies included described two or more core components of parental education interventions designed for parents of preterm infants in NICU settings. The thematic synthesis of the included studies revealed three main thematic domains: (1) educational content areas, (2) structural features of intervention programmes, and (3) parental support needs integrated within or alongside educational delivery (see Figure 2).

#### 3.1.1. Educational Content

Educational content was the most consistently reported component across the 33 studies; however, the emphasis on specific topics varied widely. Six thematic categories were identified: NICU environment, infant health, infant behaviour, infant care, parental aspects, and discharge planning (Table 1), highlighting the lack of consensus regarding what constitutes a core curriculum for NICU parental education.

NICU Environment: 21 studies (64%) addressed educational content related to the NICU environment. These included information about the physical setting, use and function of medical equipment, ward routines, staff roles, visitation policies, and infection control procedures. Although this information is widely recognised as foundational for reducing anxiety and fostering parental confidence, the depth and framing of the content differed substantially. Some interventions provided only brief descriptions of equipment and routines, whereas others offered comprehensive, context-sensitive explanations intended to demystify clinical processes and promote parents’ active involvement in care. However, few studies have articulated how this environmental orientation is expected to influence parental readiness or outcomes, revealing an important conceptual gap in the design and evaluation of such educational components.

Infant Health: Twenty-three studies (70%) included educational content focused on infant health; however, the depth and specificity of this information varied considerably. Commonly addressed topics included interpreting the infant’s medical condition, recognising symptoms and warning signs, distinguishing typical from atypical physical features of preterm infants, and managing ongoing health concerns. While the intent of this content was to support parents in understanding clinical information and participating in informed care decisions, few studies have clearly articulated how this knowledge translates into improved parental confidence or caregiving competence. This inconsistency reflects an underlying uncertainty regarding the appropriate balance between providing clinically detailed information and ensuring that it is accessible and meaningful for parents whose health literacy and emotional readiness may differ significantly. Overall, the synthesis suggests that infant health education is widely prioritised but conceptually underdeveloped, with limited integration into broader decision-making or empowerment frameworks.

Infant Behaviour: Nineteen studies (58%) sought to help parents interpret preterm infant behaviour, focusing on behavioural cues, emotional signals, sleep–wake patterns, stress indicators, and responsiveness to interaction. Although this content aligns closely with developmental care principles, the approaches used across studies varied from simple cue-recognition training to more comprehensive programmes emphasising self-regulation and co-regulation strategies. Education in this domain frequently overlaps with nurturing care techniques, such as calming strategies and cue-based engagement. However, only a minority of studies have linked behavioural-cue education to measurable outcomes, such as enhanced parental sensitivity or improved infant self-regulation, limiting the field’s understanding of the mechanisms through which behaviour-focused education may support parent–infant bonding and developmental trajectories. This gap highlights the opportunities to better embed behavioural education within coherent developmental care frameworks.

Infant Care: Infant care was a central focus of most interventions, with feeding education appearing in 24 (73% studies as the most consistently addressed component. Guidance ranged from early breastmilk expression to tube feeding and the transition to independent oral feeding, reflecting the central role of nutrition in neonatal recovery. Feeding education frequently intersects with skin-to-skin care and tactile engagement, indicating a growing recognition of the relational and physiological benefits of close physical contact. Some studies included broader caregiving practices, such as holding, cuddling, kangaroo mother care, hygiene routines, and safe handling techniques. While these components are widely understood to promote bonding and enhance parental self-efficacy, the varying depth and framing across studies suggest a lack of consensus on the essential caregiving competencies that parents should acquire prior to discharge. Moreover, few programmes explicitly considered cultural or contextual differences that may shape caregiving practices, pointing to the need for more adaptable and culturally responsive infant-care education.

Parental Aspects and Family Roles: Parental and family-related aspects were addressed in 20 studies (61%), although often as secondary or embedded elements of broader interventions rather than as stand-alone educational priorities. These components include support for maternal and paternal roles, emotional and psychological adjustment to preterm birth, parental well-being, and the promotion of secure attachment. Despite being central to parental readiness and the family’s adaptation to the NICU environment, the integration of psychosocial support has been inconsistent, with some studies offering structured guidance and others providing only minimal or implicit support. A minority of interventions acknowledged extended family members, such as grandparents and siblings, as part of the caregiving ecosystem, although this remained an underexplored area. The inconsistency in how parental and family roles were addressed highlights a conceptual gap: while emotional readiness and relational strengthening are critical to effective caregiving, these domains remain insufficiently theorised and underrepresented within NICU education programmes.

Discharge Planning and Transition to Home: Twenty-five studies (76%) addressed discharge planning and transition to home, offering education on post-discharge routines, recognising warning signs, managing medications and feeding, and navigating follow-up care. Despite being widely viewed as essential for continuity of care and parental confidence, the scope and timing of discharge-related education varied substantially. Some programmes integrated discharge planning throughout hospitalisation, while others provided it only at the final stages, potentially limiting parents’ opportunities to consolidate learning and prepare psychologically for the shift to home care. Moreover, few interventions explicitly addressed disparities in home resources, caregiver availability, or access to community health services, factors known to influence post-discharge outcomes. This suggests that while discharge education is acknowledged as important, its implementation remains uneven and insufficiently contextualised, leaving critical gaps in supporting families during the high-risk transition from the NICU to home.

#### 3.1.2. Programme Structure and Delivery

Delivery methods were described in 29 studies (88%); however, the approaches used varied markedly, reflecting differing assumptions about how parents best learn and engage in the NICU context. The interventions ranged from bedside coaching and structured group sessions to printed materials, digital platforms, and blended models. This diversity suggests an absence of consensus on optimal delivery modalities and highlights the influence of local resources, staffing structures, and parental availability in shaping programme design. While bedside coaching offers personalised, context-sensitive support, studies have seldom examined its relative effectiveness compared with group formats or digital approaches. Similarly, although digital platforms have been increasingly incorporated, few studies have evaluated their accessibility for parents with different levels of digital literacy or socioeconomic backgrounds.

The structural features of the programmes, reported in 27 studies (82%), including frequency, duration, session length, and timing, were highly inconsistent across interventions. This variability reflects the flexibility in adapting education to clinical workflow demands but also underscores the limited evidence base guiding decisions about dosage or timing. Notably, very few studies have theoretically (e.g., through adult learning principles) or empirically justified their structural choices, indicating a broader gap in how programme structure is conceptualised and evaluated within NICU education.

A total of 25 studies (76%) identified key stakeholders responsible for delivering education, most commonly nurses, developmental care specialists, occupational therapists, or multidisciplinary teams. However, few studies have explored which professional roles or combinations may be most effective or how different levels of expertise influence parents’ learning experiences. The limited attention to facilitator competencies suggests that the educator’s role is often assumed rather than critically examined, despite its likely influence on programme quality and relational engagement.

Across the literature, only a small number of studies referenced established frameworks, such as Family Integrated Care (FICare) and Family-Centred Care (FCC), each appearing in three studies, while the COPE programme was mentioned twice. Most remaining interventions were highly localised or programme-specific, indicating innovation and fragmentation within the field. The minimal uptake of established frameworks suggests that NICU education remains largely practice-driven rather than theory-informed, potentially limiting the coherence and transferability of programme designs across contexts.

#### 3.1.3. Geographic and Economic Context

This review also examined the geographic and economic contexts in which these interventions were developed. Sixteen studies were conducted in high-income countries, and four were conducted in upper-middle-income countries. A notable portion (12 studies) originated from lower-middle-income countries, of which ten were from Iran. Only one study was conducted in a low-income country (the Republic of Korea). This variation highlights both the global relevance of NICU-based parental education and the need for increased attention to intervention development in lower-resource settings (see Table 2).

Overall, while the content and structure of parental educational programmes were well described, the development processes underlying these interventions were less frequently reported. Additionally, 28 of the 33 studies included in the review evaluated the effectiveness of their programmes, indicating a strong interest in measuring outcomes such as parent confidence, stress reduction, and enhanced parent–infant interaction. These findings also help contextualise the relevance of the key components identified through the synthesis.

### 3.2. Discussion

This review synthesised evidence from 33 high-quality studies to examine how parental education programmes are conceptualised and delivered during NICU hospitalisation. Rather than identifying novel educational topics, this review aimed to clarify and synthesise core educational components across existing interventions to support greater transparency, comparability, and contextual adaptation of NICU-based parental education programmes. Three overarching domains were identified: educational content, programme structure and delivery, and parental support needs, each reflecting different assumptions about what parents require during the emotionally intense and clinically complex NICU experience. While these domains were widely represented across studies, substantial variation was evident in how educational interventions were framed, prioritised, and integrated within clinical practice, highlighting important gaps in conceptual coherence and implementation.

Within this framework, the educational content domain captured what parents were taught during NICU hospitalisation and how this knowledge was positioned to prepare them for caregiving and decision-making. Across this domain, studies consistently addressed key topics such as the NICU environment, infant health, infant behaviour, caregiving (including feeding and developmental care), parental well-being and discharge preparation. However, the thematic synthesis revealed substantial variation in the framing and depth of these topics, suggesting a lack of consensus regarding the essential curriculum elements. Although foundational knowledge is critical for parents’ confidence and readiness to participate in care, few studies have explicitly linked content choices to theoretical principles (e.g., adult learning theory, developmental care frameworks) or to empirically supported mechanisms of change. This gap limits our understanding of why specific content matters and how it contributes to parental competence, stress reduction, or infant outcomes.

Within this framework, the educational content domain captured what parents were taught during NICU hospitalisation and how this knowledge was positioned to prepare them for caregiving and decision-making. The domain of programme structure and delivery showed pronounced heterogeneity, with interventions differing in intensity, duration, timing, educator roles, and formats (e.g., bedside coaching, group sessions, and digital modules). This flexibility may enable adaptation to local workflows and resource constraints; however, the inconsistency across studies highlights the limited evidence base guiding structural decisions. Importantly, very few studies justified their structural choices or examined how specific delivery features (e.g., frequency or mode of teaching) influenced parents’ engagement or their learning outcomes. The educator role, typically performed by nurses or developmental specialists, was also rarely theorised or evaluated, despite its likely influence on relational support and parental trust. Collectively, these gaps indicate that programme structures are often practice-driven rather than evidence- or theory-driven, reducing their transferability and scalability.

In addition to content and delivery considerations, parental support needs have emerged as a critical, albeit inconsistently addressed, component of NICU educational interventions. Parental support needs have emerged as essential, yet inconsistently integrated components. While many interventions acknowledged the psychological and relational challenges faced by parents of preterm infants, only a minority explicitly embedded psychosocial or emotional support into their educational design. Programmes such as Family Integrated Care (FICare) and Family-Centred Care (FCC) illustrate more holistic models that integrate emotional, relational, and educational support, suggesting that effective parental preparation extends beyond knowledge acquisition to include confidence building, role attainment, and family empowerment. The variability in how emotional support was addressed across studies demonstrates a persistent tension between biomedical and relational models of care in NICU education.

While most of the included studies addressed elements of parental education in fragmented or variable ways, a small number of interventions provided more comprehensive and transparent accounts of programme development, contextual influences, and educational design. Three established programmes–Creating Opportunities for Parent Empowerment (COPE), FICare, and FCC–were described in sufficient detail to illustrate how educational content, programme structure, and parental support can be intentionally integrated into practice. These programmes are discussed below as illustrative exemplars rather than prescriptive models.

#### 3.2.1. Creating Opportunities for Parent Empowerment (COPE)

COPE, a structured psychoeducational intervention developed in the United States by Bernadette Melnyk in 2001, demonstrated benefits in reducing parental stress and depression, although it did not influence the length of hospital stay [32]. These findings suggest that structured educational interventions can meaningfully support parental psychological well-being during NICU hospitalisation, even when broader clinical outcomes remain unchanged. In Iran, Mianaei et al. [4] similarly reported that mothers who participated in COPE experienced lower anxiety and stress levels and demonstrated greater engagement in their infants’ care. Together, these findings indicate that psychoeducational approaches, such as COPE, may be particularly valuable in supporting parents’ emotional adjustment and confidence during the early stages of NICU admission.

#### 3.2.2. Family Integrated Care (FICare)

FICare, developed in Canada in 2013 [38], has been implemented across several settings, including Canada, Australia [50], and Spain [69]. Across the included studies, FICare was associated with increased parental confidence, enhanced role attainment, improved parent–parent communication, and more positive overall NICU experiences [38,50,69]. Broom et al. [50] highlighted improvements in parental confidence and relationship building, whereas Moreno-Sanz et al. [80] reported high parental acceptability and willingness to participate in FICare again. In addition, Benzies et al. [95] reported a reduced hospital stay duration for infants whose parents participated in FICare.

From a clinical perspective, these findings suggest that integrating parents more actively into daily care routines may support both parental well-being and service delivery. FICare exemplifies how education can be embedded within everyday caregiving activities, allowing parents to acquire knowledge and skills through supported participation rather than through discrete teaching sessions alone.

#### 3.2.3. Family-Centred Care (FCC)

Family-Centred Care (FCC), an approach aimed at minimising parent–infant separation during advanced NICU care, was found to be effective in studies conducted in China [93]. Additional evidence from India demonstrated improved parental participation in caregiving activities without adverse clinical effects, such as increased infection rates [90], as well as improvements in infant weight gain, breastfeeding outcomes, parental clinical knowledge, satisfaction, and reduced stress [3]. These findings highlight the potential of FCC approaches to promote parental involvement while ensuring clinical safety.

Both FICare and FCC extend beyond traditional parent education programmes by integrating educational content within the broader philosophies of care that prioritise relational support, partnership, and parental empowerment in the NICU [96]. Rather than positioning education as a standalone intervention, these approaches illustrate how educational components can be delivered alongside emotional and relational support, reinforcing parents’ roles in the care team.

Collectively, the findings of this review underscore an important disconnect in the literature: while outcome evaluation is common, detailed reporting of programme development, theoretical rationales, and implementation processes remains scarce. This limits the field’s ability to identify the components that contribute meaningfully to improved outcomes and constrains opportunities for replication and contextual adaptation. Moving forward, NICU education research would benefit from greater methodological transparency and alignment with conceptual frameworks that articulate how knowledge, confidence, emotional readiness, and parent–infant interactions are expected to change through educational support.

### 3.3. Limitations and Future Research

Several limitations within the existing evidence base warrant consideration. First, programme development processes were infrequently described, hindering understanding of the rationale behind content selection, structural decisions, and implementation strategies. More robust reporting, aligned with guidance such as TIDieR or intervention mapping principles, is essential for strengthening replicability and theoretical coherence.

Second, although most studies referred broadly to “parents”, the evidence remains disproportionately focused on mothers, with minimal attention paid to fathers, nonbiological caregivers, or extended family members. Given the increasing recognition of fathers’ involvement and the broader family system’s role in caregiving resilience, future research should more explicitly examine gendered experiences and family dynamics.

Third, the optimal structure of NICU educational interventions, particularly the ideal intensity, timing, and mode of delivery, remains unclear. Future studies should employ rigorous comparative designs or realist-informed approaches to identify what works, for whom, and under which conditions, particularly in resource-constrained settings.

Additionally, the exclusion of non-English full-text articles may have introduced language bias, and future reviews should consider the use of validated translation procedures or multilingual review teams to broaden inclusion.

Finally, the concentration of studies in high-income countries introduces a contextual bias. Educational interventions developed in well-resourced NICUs may not translate effectively to settings characterised by staff shortages, high patient volumes, limited parental access or socioeconomic constraints. There is a pressing need for contextually grounded research in diverse global settings, particularly in low- and middle-income countries.

## 4. Conclusions

This systematic review identified the core components, structures, and support mechanisms underpinning NICU educational interventions for parents of preterm infants. Overall, such interventions consistently contribute to enhanced parental knowledge, reduced stress, improved parent–infant interaction, and, in some settings, improved infant developmental outcomes. However, the evidence base is characterised by variable programme structures, inconsistent theoretical underpinnings, and limited reporting of development processes, making it challenging to determine which components are most critical for the impact.

The geographical concentration of evidence, predominantly from high-income countries, raises concerns about global applicability. To advance equitable neonatal care, future research must focus on designing and evaluating contextually relevant, culturally sensitive, and neuroscience-informed educational interventions in under-resourced settings. This requires attention to feasibility, cultural acceptability, caregiver diversity, and health system integration. Strengthening these areas will support the scalable adoption of parent education programmes that not only enhance parental well-being but also optimise early neurodevelopmental outcomes for vulnerable preterm infants across diverse global contexts.

## Figures and Tables

**Table 1:** Summary of educational content categories across the included studies (*n* = 33).

Authors	Country	Effectiveness	NICU Environment	Infant Health	Infant Behaviour	Infant Care	Parental Aspects	Discharge Planning
Bostanabad et al. [68]	Iran	Yes	✓	✓	✓		✓	
Bracht et al. [38]	Canada	Yes	✓	✓		✓	✓	✓
Broom et al. [50]	Australia	Yes		✓		✓		✓
Chen et al. [69]	China	Yes			✓		✓	✓
Chen et al. [70]	China, Taiwan	Yes	✓	✓	✓	✓	✓	✓
Cheng et al. [71]	China, Taiwan	Yes				✓		✓
Evans et al. [72]	Australia	No	✓			✓	✓	✓
Fotiou et al. [73]	Greece	Partial				✓	✓	✓
Gök & Efe [74]	Turkey	N/A	✓			✓		✓
Hadian et al. [75]	Iran	Yes	✓		✓	✓	✓	✓
Heo & Oh [76]	Republic of Korea	Yes	✓	✓		✓	✓	
Jafarzadeh et al. [77]	Iran	Yes	✓	✓	✓	✓	✓	
Kadiroğlu & Güdücü [78]	Turkey (Eastern Anatolia)	Yes	✓	✓	✓	✓	✓	
Khanjari et al. [79]	Iran	Yes			✓	✓	✓	✓
Lv et al. [2]	China	Yes		✓		✓		✓
Maria et al. [3]	India	Yes	✓	✓	✓	✓		✓
Mianaei et al. [4]	Iran	Yes	✓	✓	✓	✓		✓
Milgrom et al. [10]	Australia	Yes	✓	✓	✓	✓		✓
Moreno-Sanz et al. [80]	Spain	Yes			✓	✓		
Morey & Gregory [81]	USA	Yes	✓	✓	✓	✓	✓	✓
Moudi et al. [82]	Iran	Yes	✓	✓		✓	✓	✓
Mousavi et al. [83]	Iran	Yes	✓	✓		✓		✓
Nieves et al. [84]	USA	Yes	✓	✓	✓	✓	✓	
Ong et al. [85]	Malaysia	Yes		✓	✓		✓	✓
Petteys & Adoumie [86]	USA	Yes	✓	✓	✓	✓	✓	✓
Peyrovi et al. [87]	Iran	Yes			✓			✓
Phianching et al. [88]	Thailand	Yes	✓	✓	✓	✓	✓	✓
Rostami et al. [89]	Iran	Yes		✓	✓	✓	✓	
Sivanandan et al. [90]	India	N/A	✓	✓		✓		✓
Steinhardt et al. [5]	Germany	Yes		✓	✓	✓	✓	
Viera [91]	Brazil	N/A	✓			✓		✓
Yu et al. [92]	China	Yes		✓		✓	✓	✓
Zhang et al. [93]	China	Yes	✓	✓		✓		✓
			21 (64%)	23 (70%)	19 (58%)	29 (88%)	20 (61%)	25 (76%)

✓ indicates that at least one educational component within the category was reported in the study. The component-level coding is provided in the Supplementary Materials. N/A indicates that the intervention effectiveness was not measured or evaluated in the study.

**Table 2:** Distribution of countries included according to economic classification.

Low-Income Countries	Lower-Middle-Income Countries	Upper-Middle-Income Countries	High Income
Republic of Korea (*n* = 1)	India (*n* = 2) Iran (Tehran, Isfahan) (*n =* 10)	Brazil (*n =* 1) Malaysia (*n =* 1) Thailand (*n =* 1) Turkey (Eastern Anatolia region) (*n =* 1)	Australia (*n =* 3) Canada (*n =* 1) China (Taiwan) (*n =* 6) Greece (*n =* 10) Germany (*n* = 1) Spain (*n =* 1) USA (*n =* 3)

Countries are shown by their economic income according to the World Bank [94].

## Data Availability

The original contributions presented in this study are included in the article/Supplementary Materials. Further inquiries can be directed to the corresponding author.

## Supplementary Materials

The following supporting information can be downloaded at: https://www.mdpi.com/article/10.3390/children13020280/s1, Table S1: PRISMA Checklist; Table S2: Characteristics of included studies. References [97,98,99,100,101] are cited in the Supplementary Materials.

## Abbreviations

ADHD	Attention-Deficit/Hyperactivity Disorder
COPE	Creating Opportunities for Parent Empowerment
EPPI	Evidence for Policy and Practice Information
FCC	Family Centred Care
FICare	Family Integrated Care
JBI	Joanna Briggs Institute
NICU	Neonatal Intensive Care Unit
NIDCAP	Newborn Individualized Developmental Care and Assessment Program
PIOT	Population, Intervention, Outcome, Time
PRISMA	Preferred Reporting Items for Systematic Reviews and Meta-Analyses
