# Supplementary material for: Key Components of Parenting Education Interventions for Preterm Infant–Parent Dyads Admitted to the NICU: A Systematic Review
Source: Children (Basel). 2026 Feb 18;13(2):280. doi: 10.3390/children13020280 (PMC12940051; doi:10.3390/children13020280)
Supplement: Supplementary file 1 [file children-13-00280-s001.zip › Table S2. SUPPL - Characteristics of included studies.pdf]

Authors	Title	Country	Aim	Methodology	Sample	Incl/Excl criteria	Effective	Specific programme name	Educational component	Delivery	Frequency	Characteristics of teaching	Key people in educational programme
Bostana-bad (2017)	Effect of a Supportive-Training Intervention on Mother-Infant attachment.	Iran	To investigate the effect of supportive-training intervention of fathers of premature infants on mother-infant attachment	Intervention	23 parents	INCL: infants 34-37 weeks, Apgar 5 at 7 min, no congenital abnormalities, 2 days of hospitalisation, not intubated, oral feeding, primiparous mother, agree to participant and give IC. No psychological problems, meet min elementary education demanded, living with spouse.<br>EXCL: Unfortunate incident during intervention, e.g. infant death, absence/ unwillingness to continue with study.	Yes		NICU working environment & staff<br>Preterm behaviour<br>Symptoms of premature infants<br>Father's supporting role<br>Physical maternal changes<br>Emotional maternal changes<br>Observe infant<br>Take care of infant<br>Physical contact with infant	F2F individual education<br>PPT lecture<br>Q&A<br>Print<br>Digital (CD)<br>Unit visit<br>Telephone support<br>Bedside observation<br>Physical care	F2F educ: 2x 60min<br><br>F2F educ: 3 days for 30min<br><br>Obs: 30 min	Supportive teaching methods - colour manual, CD with user instructions	Content approved: neonatologist and 2 professors from nursing and midwifery researcher
Bracht (2013)	Implementing family-integrated care in the NICU	Canada	Examine the develop-ment, implementation and qualitative assessment of the education components of a family-integrated care programme	Qualitative	39 mothers	INCL: Infants born at 35 or fewer weeks' gestation	Yes	Family Integrated Care (FICare)	Infant health information (symptoms)<br>Coping, Positive parenting, Attachment, Self-care<br>Adapting to having an infant in NICU (stress)<br>Information on NICU environment & procedures<br>Infant development & care<br>Skin-to-skin holding<br>BF<br>Infection prevention (NICU procedures)<br>Discharge planning<br>Specific education:<br>BF, KMC, Baby massage<br>Bedside guidance - behavioural clues, developmentally appropriate care demonstrations, individual concerns discussed in private	Single information sessions<br>Individual support<br>Print<br>Individual psychosocial support<br>Specific education<br>Online<br>Bedside	Support: weekly	Mutual respect between families and healthcare providers<br>Positive and empathetic concern for individuality of the learner<br>Empower families to recognize and develop own abilities to meet their own needs, solve own problems, mobilize own resources to feel in control<br>Create environment conducive to learning<br>Establish privacy, limit noise and disruption, give enough time to teach<br>Introduce information slowly, allow and acknowledge accomplishment, reinforce all teaching steps<br>Set mutual goals<br>Draw on learner experiences - ask how they learn best (mode)<br>Allow learners to make mistakes<br>Evaluate teaching - ask questions, verbal and nonverbal feedback	Veteran parent mentors<br>Staff coordinator<br>Group leaders/ facilitators<br>Provided by nurses and neonatologists

Authors	Title	Country	Aim	Methodology	Sample	Incl/Excl criteria	Effective	Specific programme name	Educational component	Delivery	Frequency	Characteristics of teaching	Key people in educational programme
Broom (2017)	Exploring parental and staff perceptions of the family-integrated model	Australia	To describe parents' and staff perceptions of the benefits of each component of the FICare programme and second to explore parents' and staff perceptions of the FICare programme in an Australian NICU	Qualitative	5 parents<br>8 staff	INCL: Family member able to spend 68hr p/day with infant.<br>Admitted to unit for at least 3 weeks	Yes	Family Integrated Care (FICare)	Bedside education - KMC, understanding of infant condition and needs, developmental care, breastfeeding, infant resuscitation, and going home from hospital	Bedside<br>Physical care<br>Group<br>Ward rounds<br>Psychosocial support<br>Print<br>Diary	F2F educ: 4x p/week	Collaborative<br>Practical	NICU staff
Chen (2016)	Effect of an educational intervention on parental readiness for premature infant discharge from the NICU (2015 accepted)	China	To examine the effect of an educational intervention on parental readiness for premature infant discharge from neonatal intensive care units	Quasi-experimental	126 parents	INCL: 18 years or above; primary caregiver of premature infant after discharge; education level of grade 8 or above; home as the child's discharge destination.<br>EXCL: child experienced surgery; had a congenital malformation; or was abandoned, readmitted or deceased	Yes	The discharge education programme	Characteristics of premature infant<br>Newborn touching<br>Kangaroo care<br>Feeding<br>Bathing<br>Keeping infant warm<br>Infection prevention	2 F2F sessions (60 min each)<br>Audio-visual (videos)<br>Written (brochure)	2 sessions, 60 minutes each<br>1 week prior to discharge	In-person (Lectures)<br>Audiovisual (Videos)<br>Written (brochure containing same information as lectures & videos)	Parents<br>Presenter of education sessions not stated
Chen (2019)	The Effectiveness of an Intervention Program for Fathers of Hospitalized Preterm Infants on Paternal Support and Attachment 1 Month After Discharge	China, Taiwan	To evaluate the effectiveness of an early intervention programme to reduce paternal stress and increase fathering ability after a preterm infant's admission to the special care nursery and to influence paternal support for the mother and the father's attachment to the infant 1 month later.	Comparison	82 fathers	INCL: Fathers who had a neonate gestational age ranging from 32 to less than 37 weeks; the neonate was required to stay in the SCN for at least 5 days; and the father was living with the infant's<br>mother.<br>EXCL: Neonates were excluded from the study if they had congenital anomalies	Yes	Early fatherhood intervention programme in the special care nursery	**At admission:** NICU orientation, visiting policy, collecting, storing and transporting breastmilk.<br>**1st visit post admission**: breastmilk collection, realistic breastmilk amounts.<br>**2nd visit post admission**: infant appearance, parental interaction, infant responses.<br>**3rd visit post admission**: Developmental care in NICU.<br>**4th visit post admission:** infant behavioural cues, parental interaction with infant.<br>**Remaining parental visitations**: infant feeding, changing diapers, calming infant, paternal involvement in care.<br>**Booklet**: infant care equipment, developmental care in NICU/SCN, infant nutrition, infant appearance, parental role while infant in NICU/SCN, relaxation tips for fathers.<br>**Videotapes**: infant bathing skills, infant CPR.<br>**Handbook:** infant feeding, infant bathing.	Print<br>Individual support<br>Digital<br>Psychosocial<br>Demonstrati<br>on<br>Video	4x visitations	Prior to discharge<br>Booklet<br>Videotapes<br>Handbook	Fathers.<br>NICU nurses.

Authors	Title	Country	Aim	Methodology	Sample	Incl/Excl criteria	Effective	Specific programme name	Educational component	Delivery	Frequency	Characteristics of teaching	Key people in educational programme
Cheng (2018)	The effectiveness of learning portfolios in learning participation and learners' perceptions of skills and confidence in the mother of preterm infant	China, Taiwan	To investigate the effectiveness of preterm infant learning portfolios in enabling mothers to develop infant care knowledge and skills, as well as confidence in their abilities.	Quasi-experimental	52 mothers	INCL: Mother's (age 20 and above) of preterm infants (gestational age < 37 weeks and > 28 weeks) at the neonatal intermediate unit of a medical centre in central Taiwan.<br>EXCL: Mothers with a disability, cancer, or a psychiatric diagnosis or whose preterm infants had a congenital disorder.	Yes	The Preterm Infant Care Learning Portfolio (PICLP)	Knowing my baby include improving attachment (e.g. massage and the skin-to-skin care),<br>Breastfeeding and feeding,<br>Excretion,<br>Bathing,<br>Vaccination,<br>Sleeping,<br>Chest care,<br>Growth and development,<br>CPR and choking,<br>Body temperature,<br>Fever management	Print<br>Individualised education<br>Instruction<br>Digital<br>Demonstrations	15 min programme instruction<br>5-10 min follow-ups		NICU Nurses.
Evans (2017)	Mother-Very Preterm Infant Relationship Quality: RCT of Baby Triple P	Australia	To investigate the effects of the parenting intervention BTP for parents of very preterm infants on the quality of the mother–very preterm infant relationship and a mother's attachment and responsiveness to her very preterm infant at 6-weeks CA and 12-months CA.	RCT	120 families	EXCL: infants born with major congenital abnormalities; families who were unwilling to complete assessment requirements at 24-months CA; and parents who could not speak English.	No	Baby Triple P (BTP)		F2F in hospital<br>Telephonically<br>Audiovisual<br>Written	4 in hospital sessions.<br>4 Telephonic consultations (3 min).<br>Tip sheets every 3 months.		Mother of infant.<br>Research nurse.<br>BTP facilitators.
Fotiou (2016)	Parental stress management using relaxation techniques in a neonatal intensive care unit: A randomised controlled trial	Greece	To investigate the effect of relaxation techniques on the stress/ anxiety of parents with hospitalised premature infants, three months following discharge from the neonatal intensive care unit.	RCT	59 parents	INCL: infants <37 weeks of gestational age admitted to the same NICU, fluency in Greek, and no previous NICU experience.<br>EXCL: parents of premature infants with - birth weight of <1 kg or >2 kg, major anomalies and fatal diseases, and parents dependent on psychotropic drugs or toxic substances.	Partial		What is the NICU.<br>Needs of premature infant.<br>Parental stress.<br>Breast-feeding.<br>Why does a baby cry.<br>Taking the baby home.<br>Stress and lifestyle.<br>Physical activity and healthy diet.<br>Positive thinking.<br>Self-awareness.<br>Relaxation techniques.	Visual (PowerPoint).<br>Interactive discussions.<br>Practical exercises.<br>Audio CD.	Medical briefing daily.<br>5 sessions (90 min each).<br>Telephone calls weekly.<br>3 month reminder text messages post discharge.	Visual (PowerPoint)<br>Interactive discussions<br>Practical exercises<br>Audio CD	Parents of infant.<br>Investigator (researcher)

Authors	Title	Country	Aim	Methodology	Sample	Incl/Excl criteria	Effective	Specific programme name	Educational component	Delivery	Frequency	Characteristics of teaching	Key people in educational programme
Gök (2022)	The effect of Web-based preterm infant care training on mothers' self-confidence	Turkey	This study was conducted to examine the effect of the Web-based infant care training programme given to mothers of preterm infants on the self-confidence levels of mothers.	Quasi-experiment	84 mother-infant dyads	INCL (mothers): (a) having a preterm infant; (b) Mothers whose babies met the criteria for discharge from the hospital; (c) at least primary school graduate; (d) internet access at home or work; (g) an Internet user; (h) speaking Turkish; and (i) an agreement to join.		Web-based education programme	Preterm Infant,<br>The Importance of Breast Milk,<br>My Infant's Bath and Care,<br>My Infant's Safety<br>Communication with my Infant<br>Infant's Vaccines<br>Infant's Health Issues<br>Infant's Screening Tests<br>Developmental Recommendations<br>Lullabies for Infant	Online	4 weeks	Web-Based (DISCERN)	NICU nurses.<br>Researcher.<br>Mothers of preterm infants.
Hadian (2022)	The effect of training the fathers to support their wives on stress and self-efficacy in mothers of premature newborns hospitalized in NICU: A quasi-experimental study	Iran	To evaluate whether training the fathers to support their wives impacts premature newborn mothers' stress and self-efficacy.	Quasi-experimental	75 mothers and fathers	INCL: mothers and premature newborns in the 32–37 weeks of pregnancy, lack of newborn congenital anomaly, lack of confrontation of mothers with other stressful events during the past 6 months, the outcome of a wanted pregnancy, lack of any record of using drugs effect on the mental health of the mothers, lowest elementary school literacy, a singleton infant, and lack of hospitalization record in NICU for their previous infant.<br>EXCL: Death or discharge of the newborn from the hospital during the study and lack of parents' willingness to continue participation in the research.	Yes		**First session**: Preterm birth, Premature newborn status, Explaining roles of staff and care provided, Answering questions.<br>**Second session**: Mothers physical and mental changes, Role of fathers, Skin-to-skin contact, Proper positioning, Diaper change.	2 F2F sessions (90 min each)	2 sessions	Audiovisual (PowerPoint, videos)<br>Demonstrations (infant mannikin model)<br>Written material (booklet, 2 pamphlets)	Fathers.<br>NICU health care staff (therapists, nurses).<br>Researcher.

Authors	Title	Country	Aim	Methodology	Sample	Incl/Excl criteria	Effective	Specific programme name	Educational component	Delivery	Frequency	Characteristics of teaching	Key people in educational programme
Heo (2019)	The effectiveness of a parent participation improvement programme for parents on partnership, attachment infant growth in a neonatal intensive care unit: A randomized controlled trial	South Korea	To develop a Parent Participation Improvement Program for parents in neonatal intensive care units and to evaluate its effects on parents' partnerships with nurses, attachment to infants, and infants' body weight.	Literature review & RCT	132 infants and parents.	INCL (infants): <37 weeks gestation, receiving high-flow nasal cannula or less respiratory support, and not expected to be discharged within one month.<br>INCL (parents): willingness to spend time with the infants according to the programme's plan, completed informed consent.	Yes	Parent Participation Improvement Programme	**Stage 1**: Individualized interaction - identifying factors that impede parenting experience in NICU and establishing educational contents (successful feeding, breastfeeding, bathing, clothing, holding, knowing baby signals) for stage 2.<br>**Stage 2**: NICU environment, infant signals, infant sleep cycles, parental participation effects on infants.<br>**Stage 3:** Practical sessions covered changing diapers, breastfeeding, soothing, kangaroo care, bathing, clothing, developmental positioning, singing, and talking.		Individualized interaction stage: once. Pre-participation stage: 3 sessions. Active participation stage: 6 sessions.	Mutual goal setting and collaboration. Practical.	Parents of preterm infants. NICU nurses.
Jafarzadeh (2019)	Effect of Telenursing on Attachment and Stress in Mothers of Preterm Infants	Iran (Isfahan)	To determine the effect of telenursing on attachment and stress in the mothers of premature infants.	Clinical trail	50 mothers	INCL: Willing mothers, basic literacy, non-medical parents, stable mental/physical health, preterm infants (33-37 weeks), practicing kangaroo care, no severe disabilities, not in other studies.<br>EXCL: Unsupportive mothers, few calls, early discharge, worsening condition, infant death.	Yes	Telenursing	**Code 1**: NICU introduction and visiting policy, handwashing importance, introduction of HCPs.<br>**Code 2**: features of premature infant, physical characteristics of premature infants.<br>**Code 3**: physical changes in premature infants.<br>**Code 4**: treatments methods of premature infants (venipuncture & lumbar puncture).<br>**Code 5**: NICU equipment and their uses.<br>**Code 6**: neonatal pacification techniques.<br>**Code 7:** Various forms of infant feeding.<br>**Code 8**: expressing and storing milk.<br>**Code 9**: breastmilk increase techniques.<br>**Code 10**: relaxation music.	Telephonically	Daily (optional)	Audio (telenursing audio file)	Researcher.

Authors	Title	Country	Aim	Methodology	Sample	Incl/Excl criteria	Effective	Specific programme name	Educational component	Delivery	Frequency	Characteristics of teaching	Key people in educational programme
Kadiroğlu (2022)	Effect of Infant Care Training on Maternal Bonding, Motherhood Self-Efficacy, and Self-Confidence in Mothers of Preterm Newborns	Iran (Eastern Anatolia)	To evaluate the effect of infant care training on maternal bonding, motherhood self-efficacy, and self-confidence in mothers of preterm newborns and examine the relationship between them	Experi-mental	63 mothers and infants	INCL: Breastfed late preterm infant without high-risk issues from NICU care, with a mother who had uncomplicated childbirth, stayed at the maternal hotel, delivered at 34-36 weeks, willing to communicate, no diagnosed psychiatric illness	Yes		Infant body care.<br>Infant nutrition and feeding methods.<br>Parent-infant interaction.<br>Thermoregulation.<br>Immunization.<br>Infant home care.<br>Emotional experiences of mothers.<br>Perceptions of newborn.<br>Maternal bonding.<br>Motherhood self-efficacy<br>Maternal self-confidence	F2F training in-hospital (20 min x 4).<br>F2F reinforcement after discharge (1 x per week)	4 F2F training in-hospital.<br>F2F reinforcement after discharge once per week until infant 1 month of age.	Written (training manuals)<br>Oral presentations.<br>Visual (visual aids).	Mothers of preterm infants. NICU staff.
Khanjari (2021)	The effect of family-centered education on the quality of life of the parents of premature infants	Iran	Evaluate the comparison of the effect of family-centered education on the lives of fathers and mothers of premature infants	Quasi-experi-mental	52 parents	INCL (Parent): Proficiency in Persian, no current psychiatric/neuropsychological issues, first-time mothers, absence of recent stressful events.<br>INCL (Infant): Gestational age 30-37 weeks, birth weight ≥ 1000 g, singleton birth, no major health issues or neurological defects, no mechanical ventilation need.	Yes	Family-centred education	**First session**: Educational goals, infant hugging, breastfeeding, infant nutrition.<br>**Second session**: diaper change, bathing infant, umbilical cord care.<br>**Third session**: following up post discharge, infant vaccination, jaundice and colic of infant, principals of home care (infant sleep, infant clothing, circumcision).	F2F group sessions in-hospital	3 sessions (60 min each).	Visual (Video, presentation slides).<br>Written.<br>Practical (modelling).	Mother & father of preterm infant. Educational supervisor of the hospital.
Lv (2019)	Family-Centered Care Improves Clinical Outcomes of Very-Low-Birth-Weight Infants: A Quasi-Experiment-al Study	China	To evaluate a family-centered care intervention on clinical outcomes of very-low-birth-weight infants.	Quasi-experi-mental	319 infants	INCL: preterm infants with a birth weight <1,500g; non-invasive oxygen support; parents willing to participate in the care for at least 4 h a day.<br>EXCL: Infants with life-threatening congenital anomalies; surgery; palliative care; expected discharge within 1 week.	Yes		Basic infant care (bathing infant, changing diapers, infant temperature measurement.<br>Hygiene (when and how to wash hands).<br>Skin-to-skin contact.<br>Kangaroo care.<br>Infant communication.	In hospital F2F	Daily 4h active participa-tion.<br>Teaching session frequencies not mentioned.	Practical.	Nurses<br>Parents of infants.<br>Neonatologist

Authors	Title	Country	Aim	Methodology	Sample	Incl/Excl criteria	Effective	Specific programme name	Educational component	Delivery	Frequency	Characteristics of teaching	Key people in educational programme
Maria (2021)	Assessment of feasibility and acceptability of family-centered care implemented at a neonatal intensive care unit in India	India	To examine the feasibility and acceptability of the FCC model in a NICU in India.	Pros-pective cohort	333 parent-attendant/infant dyads, 24 doctors, 21 nurses	INCL: Parent-attendant/infant dyads, infant hemodynamically stable (without inotropes or ventilation), parent-attendant willing and available to provide care.<br>EXCL: infant unstable hemodynamically, ventilated infants, and unwilling/unavailable parent-attendant.	Yes	Family-Centered Care (FCC)	**Session 1: Sensitization to Family-Centered Care**<br>• Describing the programme<br>• Preparing for entry into nursery (handwashing, gowning, infection prevention, familiarizing with nursery environment)<br>**Session 2: Developmentally Supportive Care**<br>• Minimizing noise, holding infant, nesting, calming infant<br>• Cleaning of soiled infant<br>• Breastfeeding<br>• Expression of breastmilk<br>• Spoon/cup feeding<br>• Identifying danger signs and when to alert the healthcare provider<br>**Session 3: Kangaroo Mother Care**<br>• Upright positioning of infant<br>• Providing skin-to-skin contact<br>**Session 4: Preparation for Discharge and Care at Home**<br>• Preparing for discharge and care at home<br>• Handwashing/ prevention of infection hygiene<br>• Sponging/cleaning<br>• Appropriate clothing/thermal care<br>• Exclusive breastfeeding and skin-to-skin contact<br>• Caring of cord and eyes<br>• Identifying danger signs and when to seek medical care<br>• Following up and complying with discharge instructions<br>• Immunization	In hospital F2F.	Parent attendant - daily (optional). HCPs - prior to intervention; thereafter every 3 months.	Audio-visual training tool	Principle investigator (HCPs training sessions).<br>HCP (parent-attendant training sessions).

Authors	Title	Country	Aim	Methodology	Sample	Incl/Excl criteria	Effective	Specific programme name	Educational component	Delivery	Frequency	Characteristics of teaching	Key people in educational programme
Mianaei (2014)	The effect of Creating Opportunities for Parent Empowerment programme on maternal stress, anxiety, and participation in NICU wards in Iran	Iran	To perform the Creating Opportunities for Parent Empowerment (COPE) programme for Iranian mothers and evaluate its effectiveness on stress, anxiety, and participation of mothers who have premature infants hospitalized in neonatal intensive care units (NICUs).	RCT	90 mothers	INCL (Mothers): aged 18 or older, proficient in Persian, and with infants meeting specific criteria.<br>INCL (Infants): gestational age of 26-34 weeks, birth weight below 2500 g, and other health conditions.<br>EXCL: non-visiting mothers for over 4 days, infants who died or were discharged before completing the COPE programme.	Yes	Creating Opportuni-ties for Parent Empower-ment (COPE)	**Phase 1:** Infant physical and behavioural features, differences between mature and immature infants, NICU information, strategies to aid parents to participate in infant care.<br>**Phase 2**: Infant behaviours, infant growth and development, maternal participation strategies, taking care and meeting infant needs.<br>**Phase 3**: Infant states and behaviour, infant alertness, sleepiness, infant communication. Parental role in transitioning of infant from hospital to home. Continuation of mother-infant positive interaction. Calming infant.<br>**Phase 4**: Parental role in infant development and growth. Increasing mother-infant positive interaction.<br>Improving infant cognitive development.	Phase 1 - 3 delivered in hospital. Phase 4 delivered at home.	4 sessions. Booklet and audiotape received after each session.	Printed (workbook activities).<br>Audio (audiotapes).<br>Visual (pictorial booklet).	Mother of infant.<br>Researcher.
Milgrom (2013)	Early communication in preterm infants following intervention in the NICU	Australia	To evaluate the impact on early developmental milestones of an enhanced MITP (PremieStart) delivered over an extended period in the NICU	RCT	109 mothers	EXCL: i) insufficient spoken and written English, ii) triplets or higher multi- ples, iii) infants with congenital abnormalities, iv) infants/mothers judged to be too severely medically ill to participate by their attending physicians, v) maternal drug and alcohol abuse/dependence or vi) residing >100 km from Melbourne	Yes	PremieStart	Recognizing signs of infant stress,<br>Alert-available behaviour,<br>Facial expressions,<br>Quality of motor behaviours,<br>Posture and muscle tone,<br>Graded stimulation,<br>Avoid overwhelming infant.<br>Infant touch, movement and massage.<br>Kangaroo care,<br>Multi-sensory stimulation,<br>Debriefing,<br>Normalizing parental feelings,<br>Challenging dysfunctional thoughts,<br>Diary keeping.	F2F sessions in NICU<br>F2F session at home	Weekly 1h sessions over 9 weeks in hospital<br>1 follow-up session at home.	Interactive discussions	Psychologists.

Authors	Title	Country	Aim	Methodology	Sample	Incl/Excl criteria	Effective	Specific programme name	Educational component	Delivery	Frequency	Characteristics of teaching	Key people in educational programme
Moreno-Sanz (2021)	Scaling Up the Family Integrated Care Model in a Level IIIC Neonatal Intensive Care Unit: A Systematic Approach to the Methods and Effort Taken for Implementation	Spain	To scale up and adapt FICare to make it suitable in level IIIC NICUs, which care for extreme prematurity and other complex medical or surgical neonatal conditions.	Pilot	76 parents and 91 infants.	INCL (Infant): Infants with birth weight at or below 1,500 g or gestational age at or below 34 weeks, anticipated NICU stay for ≥ 3 weeks due to peri-neonatal conditions, and a decision for full life support were included.<br>EXCL (Infant): Infants not receiving full life support, with critical illnesses unlikely to survive, or scheduled for early transfer to another hospital were excluded. INCL (Parent): Parents willing to spend ≥ 6 hours daily in NICU, attend educational sessions, actively care for infants for ≥ 21 days, with no communication barriers, and who provided informed consent were included. EXCL (Parent): Parents with intellectual barriers hindering learning, using unintelligible language, refusing informed consent, or experiencing mental health issues or legal supervision were excluded.	Yes	Family Integrated Care (FICare)	**Healthcare workers:** Boundaries of FICare, promoting FICare, psychosocial needs of families, communication skills, family involvement in the NICU, professional self-care.<br>**Caregivers/parents**:<br>Description of FICare model,<br>introduction to the NICU,<br>family self-care,<br>infants neurobehaviors,<br>infant stress,<br>infant pain,<br>basic level infant care,<br>advanced level infant care,<br>social resources available,<br>preparing for home,<br>hand hygiene,<br>infant bathing,<br>breastfeeding,<br>other forms of feeding,<br>skin-to-skin contact,<br>kangaroo method,<br>dressing and diaper change,<br>oral medication,<br>infant body temperature,<br>infant mouth and skin care,<br>infant positioning,<br>non-invasive respiratory support,<br>urinary catheter care,<br>ostomy care,<br>daily balance,<br>invasive respiratory support.	HCPs: F2F meetings & workshops. Parents: F2F cotside & workshops.	Parents: workshops 3 x per week (45min). Cotside F2F frequency not given. Daily data recordings.	Individualized theoretical and practical learning. Printed (educational manual). Electronic (educational manual). Written (parent notebook for daily recordings).	NICU staff. Parents of infants. Project leader. Clinical staff coordinator. Social worker. Psychologist. Speech therapist. Sociologist.

Authors	Title	Country	Aim	Methodology	Sample	Incl/Excl criteria	Effective	Specific programme name	Educational component	Delivery	Frequency	Characteristics of teaching	Key people in educational programme
Morey (2012)	Nurse-Led Education Mitigates Maternal Stress and Enhances Knowledge in the NICU	USA	To evaluate the effect of a nurse-led intervention pertaining to the experience of having a baby in the NICU on maternal stress in a population of high-risk pregnant women at three different time points.	Repeated measures	42 pregnant women	INCL: (1) English literacy that encompassed speaking, writing, and com-prehension, and (2) stable clinical status, which allowed attendance and participation in the entire NICU class.<br>EXCL: Participants who enrolled in the study but did not deliver a premature baby requiring admission to the NICU.	Yes		Introduction to NICU multidisciplinary team.<br>Physiological differences of premature babies compared to full term babies.<br>Physical needs of NICU infants.<br>Comfort measures for infant.<br>Importance of breast milk.<br>Introduction to pumping breast milk.<br>NICU infection control measures.<br>Developmental care measures in NICU.<br>Role of parents during NICU admission.<br>Discharge planning and procedures.	Group classes (demonstra-tions, videos, discussions)	Weekly classes in hospital	Audiovisual (educational video).<br>Demonstra-tion (NICU tour).<br>Verbal (teaching discussion)	NICU staff.<br>Mothers.
Moudi (2019)	The effect of a care pro-gramme and social support on anxiety level in mothers of late preterm infants in Sistan and Baluchestan, Iran	Iran	To determine the effectiveness of a care pro-gramme on the anxiety level of mothers with LAMP babies and to determine the effectiveness of the care programme on the level of anxiety of new mothers in the presence of social support.	Quasi-experi-mental	79 mothers	Infant Inclusion: Singleton birth, no congenital abnormalities. Mother Inclusion: Literate, proficient in Farsi, no history of preterm birth, mental illness, or illicit drug use during pregnancy. Mothers Exclusion: postpartum psychosis, missed educational sessions, infant critical conditions (e.g., septicaemia), or neonatal death during NICU stay.	Yes		Session 1: NICU environment. Infants physical condition. Encouraging mother and infant contact. Handwashing.<br>Session 2: Breast pumping, expressing milk, collecting and storing breastmilk, feeding infant with syringe/dropper, carrying the baby, changing the diaper.<br>Session 3: Breastfeeding positions and practices, Kangaroo care.<br>Session 4: Infant massage, bathing the infant, post-discharge care and follow-up, calming the infant.	Individually F2F in NICU.	4 sessions (60 - 90 min)	Printed (pamphlets), Audio (CD's), Practical demonstrations & exercises.	Mothers.<br>Researcher.<br>NICU nursing staff.
Mousavi (2021)	Impact of Maternity Support Program on the Stress of Mothers in the First Encounter with the Preterm Infants	Iran (Tehran)	To investigate the impact of maternity support pro-gramme on the stress of mothers in the first encounter with infants.	Experimental	143 parents	INCL: newborns under 37 weeks, birth weight under 2500g, likelihood of survival, willingness to participate, Iranian nationality, and verbal communication ability.<br>EXCL: abnormality or serious conditions like intraventricular haemorrhage (IVH) grade 3 or 4.	Yes		Spiritual support.<br>Emotional support.<br>Expressing breastmilk, using a breast pump, storing and transporting expressed milk.<br>Mother-infant interaction (bedside).<br>NICU staff, equipment, and environment.<br>Information on infant characteristics and condition.<br>Infant massage.<br>Preparing mothers for the physical characteristics of premature infant.	Outside of NICU (photo albums, practical training, spiritual and emotional support).<br>Inside NICU (NICU environment training, bedside interaction with infant).<br>DVD and booklet given to be taken home.	Photo albums, practical training, emotional and spiritual support presented once.<br>DVD and booklet taken home by participant.	Supportive, interactive, and practical.<br>Visual (photos), Printed (booklet), Electronic (DVD) material	Researcher.<br>Mothers.<br>NICU staff.

Authors	Title	Country	Aim	Methodology	Sample	Incl/Excl criteria	Effective	Specific programme name	Educational component	Delivery	Frequency	Characteristics of teaching	Key people in educational programme
Nieves (2021)	Effect of a Parent Empowerment Program on Parental Stress, Satisfaction, and Length of Stay in the Neonatal Intensive Care Unit	USA	To explore the effect of implement-ing the COPE programme on parental stress, postpartum depression, parental satisfaction with care, and length of stay in a community-based hospital.	Quasi-experimental	49 parent sets	INCL: Parents at least 18 years of age, could read and write English, and had a baby born at the study institution at 35 weekś gestation or less. EXCL: infant had a lethal congenital anomaly or they had a previous child in the NICU	Yes	Creating Opportuni-ties for Parent Empowerment (COPE)	**1st phase:** Helping your premature baby grow and develop - parental feelings of stress, sadness, and loss is acknowledged and the following topics are discussed: characteristics and behaviours of premature infants, tips on interacting with infant, contrast to appearance and behaviours of full-term infants. **2nd phase:** Helping yourself and your baby during early NICU days - Education on difficulties of having an NICU infant, importance of parental self-care, infant characteristics, stress cues, infant ability to interact. **3rd phase:** Getting ready to go home together - Discussions surround infant developmental milestones, premature infants' states of awareness, parental rest and relaxation. **4th phase:** Adjusting to life at home with your baby - Infant development activities, general parenting guidelines.			In-person and practical (bedside) Written [a COPRE for HOPE book & parental journaling]	NICU nurses Principal investigator Infant parents
Ong (2019)	The effectiveness of a structured nursing intervention programme on maternal stress and ability among mothers of premature infants in a neonatal intensive care unit	Malaysia	To investigate the effectiveness of a structured nursing intervention programme on maternal stress and NICU-related maternal ability after the admission of premature infants to a neonatal intensive care unit (NICU).	Quasi-experimental	216 mothers	INCL: (a) at least 18 years of age, able to give their consent and understand either in English or Malay; (b) having premature infants between 27– 34 weeks' gestation, have an Apgar score of >5; and (c) lacking other major health problems. EXCL: Mothers who had quadruplets or more, illnesses or problems such as psychiatric disorders, maternal drug or substance abuse as identified from their medical records, infants with any surgical problems and terminally ill infants.	Yes	Structural nursing intervention programme	**First meeting:** Booklet given to parents and building of rapport (no educational components) **Second meeting:** NICU equipment, information about infants condition, relaxation techniques, answering of parental questions. **Booklet:** information about the things parents of premature infants must know: the equipment used on the baby, the baby's developmental care in the NICU, the baby's nutrition, the baby's appearance, what the baby is doing, what the parents can do with their preterm baby when they are in the NICU and relaxation tips for parents.	In person meetings. Over the telephone.	2 meetings 2 follow up telephone calls	Printed (information booklet). Audio (telephone calls) In-person (meetings)	Researcher Parents

Authors	Title	Country	Aim	Methodology	Sample	Incl/Excl criteria	Effective	Specific programme name	Educational component	Delivery	Frequency	Characteristics of teaching	Key people in educational programme
Petteys (2018)	Mindfulness-Based Neurodevelopmenta l Care	USA	To examine the impact of parent education and participation in mindfulness-based neurodevelopmenta l care on parent outcomes (stress, bonding, and satisfaction) and infant length of stay (LOS).	RCT - Pilot study	60 parents	INCL: birth gestational age <35 weeks and expected LOS >14 days, parents agreeable to spend a minimum of 1 hour at infants' bedside weekly, English-speaking). EXCL: parents unable to complete writ- ten forms and communicate in the English language were excluded.	Yes		**One-on-one educational session (30 to 60 minutes):** that taught mindfulness techniques and structured neurodevelop-mental care training activities within 10 days of enrolment and after completion of initial PSS:NICU forms. Mindfulness techniques education consisted of focused breathing, personal awareness and nonjudgment, and awareness and nonjudgment of their infant. Principles of attunement and varied types of touch and non-touch interactions were reviewed. Neurodevelopmental care training centered on observation and recognition of infant cues; signs of organized and disorganized physiologic state, motor behaviours, and state behaviours, as well as development of infant self-regulation, were reviewed.	In person	Single education session (30 - 60 min) Verbal contact every other week.	In person education (one-on-one bedside) Educational booklet (printed) Verbal contact every second week.	Researcher Parents
Peyrovi (2016)	The effect of empowerment programme on ''perceived readiness for discharge'' of mothers of premature infants	Iran	To examine the effect of empowerment pro- gramme on ''perceived readiness for discharge'' of mothers of premature infants at the time of discharge.	Quasi-experimental	80 mothers and preterm infants	INCL (mothers): age over 18 years, reading and writing literacy, not having an infant with a history of hospitalization in NICU in the past, no physical illness disrupting everyday activities and no obvious neuro- logical and psychiatric disorders that lead to drug use, and scores less than 7 in terms of readiness for discharge. INCL (infants): gestational age of 28– 34 weeks, birth weight less than 2500 grams, single born and absence of any congenital abnormality. EXCL: Discharging infant before the end of intervention, the absence of mother in every stage of the intervention and infant death were considered as exclusion criteria.	Yes	Empowerme nt plan for mothers of premature infants	NICU environment & equipment. The appearance, characteristics, and behaviours of premature infants. The sleep–awake patterns of premature infant. Premature infants signs of stress and stress relief. Parental caregiving (roles and responsibilities). Infant interaction. Proper principles and methods of daily routine care for a premature infant. NICU environment. Premature infant characteristics. Sleep-awake patterns. Signs of stress & stress relief. Parental role. Infant interaction. Daily routine. Maternal role in infant discharge. Establishing a relationship with infant. Infant healthcare (screening, vaccinations, medications, etc.). Contacting NICU / specialists.	In person	3 Sessions 0.5 - 1h each session<br><br>Sessions conducted: Session 1: three to four days after birth Session 2: two to four days after first session Session 3: one to three days prior to discharge	In-person (discussions, practical, presentations) Printed (educational supplement booklet).	Mothers Researcher Matron of NICU NICU nurses

Authors	Title	Country	Aim	Methodology	Sample	Incl/Excl criteria	Effective	Specific programme name	Educational component	Delivery	Frequency	Characteristics of teaching	Key people in educational programme
Phianching (2020)	Effects of the parental sensitivity intervention among mothers and fathers of preterm infants: A Quasi-experimental study	Thailand	To examine the effects of a parental sensitivity intervention on attachment and confidence/self-efficacy among mothers and fathers of preterm infants.	Quasi-experimental	Mothers, Fathers and Preterm infants	INCL (parents): preterm infant's biological mother and father lived together, were able to communicate in Thai, and resided in the municipality.<br>INCL (Infant): gestational age between 34-37 weeks, singleton birth and stable vital signs with no requirement for respiratory support.	Yes	Parental sensitivity intervention (PSI)	Preterm infant characteristics.<br>Infant cues and behaviours with appropriate parental response.<br>Observing, understanding, and responding to infant cues.<br>Daily infant care activities.<br>Infant-parent reciprocal behaviours.	In-person<br>Online	4 sessions (40-60 minutes each)<br>Session 1 & 2: day 1<br>Session 3: day 2<br>Session 4: day 3	In-person (discussions, practical, presentations)<br>Printed (educational supplement booklet).<br>Online (handbook).<br>Audiovisual (YouTube videos)	Parents<br>Researcher<br>NICU nurse
Rostami (2020)	Preterm infant neurodevelopmental care training programme and mother- infant attachment	Iran	Investigate the effect of neurodevelopmental care training pro-gramme for mothers with preterm infants on mother-infant attachment at one month's age.	RCT - multi-center	72 mother-infant dyads	INCL: 28-34 week of the gestational age, having a birth weight of <2500 g, having reading and writing literacy and ability to speak Persian, and being a resident of Tehran Province.<br>EXCL: absence of intraventricular haemorrhage grade III and IV, hydrocephaly, the retinopathy of prematurity grade 3 or higher, and NICU hospitalization <1 week (to have enough time to train the mothers).	Yes	The Neurodevelopmental Care Program for Mothers With Preterm Infants in the NICU	The interventions included the components of neurodevelopmental care (i.e., caring for the environment, noise, touch, smell, light, taste, sleep, movement, and position, deep sleep, stress and pain reduction, skincare, nutrition and breastfeeding, and temperature). Furthermore, various information was provided, including familiarity with the NICU environment and preterm infant, the identification of the symptoms of infant stress and the consequences of prematurity, skill training in emotional support for infants, and the acquisition of the breastfeeding-kangaroo mother care skill.<br>Educational interventions for/to:<br>1 Environmental noise reduction<br>2 Environmental light reduction<br>3 Olfactory and gustatory senses<br>4 Provide safe and restful sleep<br>5 Move, position, and manipulate the infant<br>6 Reduce pain and stress<br>7 Skin care and Kangaroo-Mother Care<br>8 Optimize nutrition<br>9 Recognize the stress behaviour<br>10 Parental communication and interaction with existing conditions and receive support and necessary information<br>11 Familiarity with preterm infant and the consequences of prematurity<br>12 Post-discharge care interventions	Education	12x sessions		

Authors	Title	Country	Aim	Methodology	Sample	Incl/Excl criteria	Effective	Specific programme name	Educational component	Delivery	Frequency	Characteristics of teaching	Key people in educational programme
Sivanandan (2021)	Implementing Family- Centered Care in the Neonatal Intensive Care Unit – A Quality Improvement Initiative	India	To implement family-centered care (FCC) in neonatal intensive care unit (NICU).	Quality improvemen t	505 neonates	INCL: Availability of at least one family member, preferably the mother for at least 6 h/d and a stable neonate based on physiological criteria irrespective of gestational age. The neonate was considered stable if less than two parameters of TOPS (Temperature, Oxygen saturation, Perfusion, Sugar) score were abnormal<br>EXCL: The neonates were excluded from FCC if they required non-invasive respiratory support (other than blended oxygen by cannula), had major congenital malformations or were admitted for comfort care.		Family-Centered Care (FCC)	**Videos on:**<br>- entry into NICU (information about do's and don'ts before entry, learning steps of hand washing and wearing a gown)<br>- familiarization with the NICU environment;<br>- discharge preparedness and care at home.<br>**Hands-on training:** provided to mothers tailored to their learning capacity and neonatal needs. Training comprised demonstration of caregiving activities (diaper-change, orogastric tube feeding and paladai feeding) by the bedside nurse followed by supervised practice.	Digital Individualise d training<br>Demonstrati on<br>Supervised bedside practice<br>Independent care	Continuous videos<br>Hands-on as required	The key interventions were:<br>(1) adoption of a unit protocol for FCC with expanded visitation hours;<br>(2) parental education through audio-visual aids, and<br>(3) capacity building through training and peer support.	Three nursing officers and two senior residents led this QI project supervised by two neonatologists.
Steinhardt (2015)	Influences of a dedicated parental training programme on parent–child interaction in preterm infants	Germany	To investigate influences on the interaction between preterm infants and their parents by a dedicated parental training pro-gramme on the care of preterm infants	Surveys	50 mother-infant dyads	INCL: Mothers of infants with a birth weight b 1500 g (VLBWI) and adequate German language skills; Approval for analyzing the videotaped behaviour.<br>EXCL: mothers with a psychiatric diagnosis and children with severe neonatal complications or non-viable infants.	Yes		Infant handling<br>Stimulation<br>Feeding and breastfeeding<br>Personal hygiene<br>First aid<br>Early development	Formal group education (8-16 parents)<br>Bedside supervised care	6x 45min over 3 weeks (2x p/week)<br>5h supervision	9 h of training, consisting of theoretical and practical parts.	Healthcare workers, Psychologists<br>Specially-trained nurses.

Authors	Title	Country	Aim	Methodology	Sample	Incl/Excl criteria	Effective	Specific programme name	Educational component	Delivery	Frequency	Characteristics of teaching	Key people in educational programme
Viera (2016)	Educative practice and maternal stress of premature infant: randomized clinical trial	Brazil	Abstract in English - article in Portuguese.<br>The aim of this study was to compare the stress levels of mothers of premature newborns during hospitalization and after hospital discharge, correlating them with the levels detected in the analysis of maternal knowledge about premature infant care.	Literature review<br>Development	46 mothers	INCL: Preterm under 37 weeks of gestational age; preterm without malformation; teenager mother following with law responsible; literacy mother.<br>EXCL: Preterm left for adoption; mothers with related psychiatric problems; mothers who auto relate that are in continuous use of controlled drugs (anxiety or depression); mothers with medical complications or who have gone to death during the study; illiterate mothers; teenagers mothers without law companion; mothers who did not attend the consultations on the follow-up clinic between the 3rd and 6th month after discharge from the Neonatal Intensive Care Unit.			Two educational simulation activities on consecutive days lasting one and a half hours each, about care for the preterm newborn (intercurrences and warning signs, breastfeeding and pumping, hygiene and diaper changing), planned according to Freire's Theory,	F2F (PPT)<br>Conversation with HCP<br>Demonstration<br>Simulation<br>Practice on doll<br>Bedside care	40min Lecture & conversation<br>10min demonstration<br>20min simulation	Practical (simulated NICU environment, infant practice dummies).<br>Verbal (conversation circles, discussions).<br>Visual (photography).<br>Printed (preterm care book).	

Yu (2022)	Sensitivity Training for Mothers With Premature Infants: A Randomized Controlled Trial	China	Enhancing Chinese mothers’ sensitivity towards their premature infants’ physiological and social cues.	RCT - double bind, parallel	60 mothers	EXCL: (a) insufficient spoken and written Chinese ability of the mother; (b) mother under 18 years of age; (c) triplets or higher multiples; and (d) infants with congenital abnormalities (e.g., metabolic disorder, chromosomal disorder).	Yes	Parent Sensitivity Program modified version of the Mother–Infant Transaction Program (MITP)	**Session 1**: Understanding the characteristics of the premature infants<br>• Using the new-born behavioural observation scale to demonstrate her own infant’s uniqueness, potential for self-regulation and interaction<br>• Photo or video illustrations of infants’ signs of overstimulation, their “shut-down” mechanisms and ways to avoid overstimulating them<br>**Session 2**: Understanding premature infants’ behavioural states<br>• Introducing infants’ different behavioural states, their changes and how infants organize their behavioural states<br>• Ways for parents to help their infants to achieve better organization<br>**Session 3:** Social interaction: engaging infants and sustaining an interaction<br>• Understanding infants’ engagement and disengagement cues<br>• What parents could do to engage their infants to optimize social interaction<br>• Recognizing different cues to facilitate daily care such as feeding and sleep<br>**Session 4:** State modulation for infants<br>• Understanding the sleep-wake cycle of infants<br>• Learning about infants’ crying management and their consolability<br>• Building a secure attachment relationship with an infant recognizing premature infant’s characteristics (e.g., skin colour changes, breathing, temperature control, motor functions, sleep), understanding and recognizing signs of infant stress, and infant’s engagement and disengagement cues, principles of graded stimulation, and how to optimize interactions and avoid over-stimulating the infant. Daily caregiving routines and the post discharge home visits in the original protocol were dropped in this modified version.	Individual coaching	4x sessions over 3 weeks in NICU	The training was delivered mainly in the format of didactic information sharing, with brief handouts (two to three pages of notes on the important concepts and information covered in the training, written in Chinese at primary school reading level) provided after each session. Nonetheless, the coaches would illustrate the concepts and techniques with the infant present at the training session. Behavioural homework was assigned to encourage the mothers to practise the skills on their infants in between sessions.	

Authors	Title	Country	Aim	Methodology	Sample	Incl/Excl criteria	Effective	Specific programme name	Educational component	Delivery	Frequency	Characteristics of teaching	Key people in educational programme
Zhang (2018)	Involvement of Parents in the Care of Preterm Infants: a Pilot Study Evaluating a Family-Centered Care Intervention in a Chinese Neonatal ICU	China	To evaluate the effectiveness and safety of a Family-Centered Care (FCC) intervention in a Chinese Neonatal Intensive Care Unit (NICU).	RCT - pilot	61 infants<br>110 parents	INCL: parents of infants born at <37 weeks of gestation, able to commit spending a minimum of four hours per day with their infant between office hours to enable attendance at medical rounds and education sessions.<br>EXCL (Infants): 1) major life-threatening congenital anomaly; 2) critical illness and unlikely to survive; 3) respiratory support (CPAP, mechanical ventilation, high-frequency oscillatory or jet ventilation, extra-corporeal membrane oxygenation).<br>EXCL (Parents): health, family, social, or language issues that might limit their integration and collaboration with the healthcare team.	Yes	Family-Centered Care (FCC)	Hand hygiene,<br>Neonatal resuscitation,<br>Daily nursing care (including bathing, feeding, and massage),<br>Respiratory support.	Education theory<br>Education practice<br>Q&A<br>Bedside supervised care	4 hours p/day<br>Education 90min p/day, 5 days/week<br>Primary care min 4 h/day	Theoretical.<br>Practical.	
