# Supplementary material for: Key Components of Parenting Education Interventions for Preterm Infant–Parent Dyads Admitted to the NICU: A Systematic Review
Source: Children (Basel). 2026 Feb 18;13(2):280. doi: 10.3390/children13020280 (PMC12940051; doi:10.3390/children13020280)
Supplement: Supplementary file 1 [file children-13-00280-s001.zip › SUPPL 1 - Summary of quality assessment of selected studies (updated).pdf]

*Supplement: Summary of quality assessment of selected studies**

No	Author	Title	Tool name	Consensus Score	I/E
1	Bostanabad (2017) [68]	Effect of a Supportive-Training Intervention on Mother-Infant Attachment	JBI Quasi-experimental	9/9	I
2	Bracht (2013) [38]	Implementing family-integrated care in the NICU: A parent education and support program	JBI Quali	6/10	I
3	Broom (2017) [50]	Exploring Parental and Staff Perceptions of the Family-Integrated Care Model: A Qualitative Focus Group Study	JBI Quali	7/10	I
4	Chen (2016) [69]	Effect of an educational intervention on parental readiness for premature infant discharge from the neonatal intensive care units	JBI Quasi-experimental	8/9	I
5	Chen (2019) [70]	The Effectiveness of an Intervention Program for Fathers of Hospitalized Preterm Infants on Paternal Support and Attachment 1 Month After Discharge	JBI Quasi-experimental	9/9	I
6	Cheng (2018) [71]	The effectiveness of learning portfolios in learning participation and learners' perceptions of skills and confidence in the mother of preterm infant	JBI Quasi-experimental	8/9	I
7	Evans (2017) [72]	Mother-Very Preterm Infant Relationship Quality: RCT of Baby Triple P	JBI RCT	23	I
8	Dibley (2016) [97]	The feasibility of using "PremieStart," a mother-premature infant interaction program, on a neonatal unit in England	MMAT	3/7	E
9	Fotiou (2016) [73]	Parental stress management using relaxation techniques in a neonatal intensive care unit: a randomised controlled trial	JBI RCT	14	I
10	Givrad (2021) [98]	Promoting infant mental health in the neonatal intensive care unit (NICU): A review of nurturing factors and interventions for NICU infant-parent relationships	JH non-research	4	E
11	Gök (2022) [92]	The effect of Web-based preterm infant care training on mothers' self-confidence	JBI Quasi-experimental	5/9	I
12	Hadian (2022) [74]	The effect of training the fathers to support their wives on stress and self-efficacy in mothers of premature newborns hospitalized in NICU: a quasi-experimental study	JBI Quasi-experimental	8/9	I
13	Helmer (2021) [99]	An Early Collaborative Intervention Focusing on Parent-Infant Interaction in the Neonatal Period. A Descriptive Study of the Developmental Framework	JBI Quasi-experimental	1/9	E
14	Heo (2019) [75]	The effectiveness of a parent participation improvement program for parents on partnership, attachment infant growth in a neonatal intensive care unit: a randomized controlled trial	JBI RCT	19	I
15	Jafarzadeh (2019) [76]	Effect of telenursing on attachment and stress in mothers of preterm infants	JBI RCT	14	I
16	Kadiroğlu (2022) [77]	Effect of Infant Care Training on Maternal Bonding, Motherhood Self-Efficacy, and Self-Confidence in Mothers of Preterm Newborns	JBI RCT	21	I
17	Khanjari (2021) [78]	The effect of family-centered education on the quality of life of the parents of premature infants	JBI Quasi-experimental	9/9	I
18	Lv (2019) [2]	Family-Centered Care Improves Clinical Outcomes of Very-Low-Birth-Weight Infants: A Quasi-Experimental Study	JBI Quasi-experimental	7/9	I
19	Maria (2021) [3]	Assessment of feasibility and acceptability of family-centered care implemented at a neonatal intensive care unit in India	JBI Cohort	6/11	I
20	Mianaei (2014) [4]	The effect of Creating Opportunities for Parent Empowerment program on maternal stress, anxiety, and participation in NICU wards in Iran	JBI RCT	20	I
21	Milgrom (2013) [10]	Early communication in preterm infants following intervention in the NICU	JBI RCT	25	I

22	Moreno-Sanz (2021) [79]	Scaling Up the Family Integrated Care Model in a Level IIIC Neonatal Intensive Care Unit: A Systematic Approach to the Methods and Effort Taken for Implementation	MMAT	4/7	I
23	Morey (2012) [80]	Nurse-led education mitigates maternal stress and enhances knowledge in the NICU	JBI Quasi-experimental	5/9	I
24	Moudi (2019) [81]	The effect of a care program and social support on anxiety level in mothers of late preterm infants in Sistan and Baluchestan, Iran	JBI Quasi-experimental	9/9	I
25	Mousavi (2021) [82]	Impact of Maternity Support Program on the Stress of Mothers in the First Encounter with the Preterm Infants	JBI Quasi-experimental	8/9	I
26	Nieves (2021) [83]	Effect of a Parent Empowerment Program on Parental Stress, Satisfaction, and Length of Stay in the Neonatal Intensive Care Unit	JBI Quan	7/9	I
27	Nourani (2019) [100]	A Smart Phone Application for the Mothers of Premature Infants	JBI Quasi-experimental	3/9	E
28	Ong (2019) [84]	The effectiveness of a structured nursing intervention program on maternal stress and ability among mothers of premature infants in a neonatal intensive care unit	JBI Quasi-experimental	8/9	I
29	Petteys (2018) [85]	Mindfulness-Based Neurodevelopmental Care: Impact on NICU Parent Stress and Infant Length of Stay; A Randomized Controlled Pilot Study	JBI RCT	13	I
30	Peyrovi (2016) [86]	The effect of empowerment program on "perceived readiness for discharge" of mothers of premature infants	JBI Quasi-experimental	7/9	I
31	Phianching (2020) [87]	Effects of the parental sensitivity intervention among mothers and fathers of preterm infants: A Quasi-experimental study	JBI RCT	7	I
32	Platonos (2018) [101]	Integrated family delivered care project: Parent education programme	JBI Quali	2/10	E
33	Rostami (2020) [88]	Preterm infant neurodevelopmental care training program and mother- infant attachment	JBI RCT	13	I
34	Sivanandan (2021) [89]	Implementing Family- Centered Care in the Neonatal Intensive Care Unit – A Quality Improvement Initiative	JBI Quasi-experimental	6/9	I
35	Steinhardt (2015) [5]	Influences of a dedicated parental training program on parent-child interaction in preterm infants	JBI Quasi-experimental	7/9	I
36	Viera (2016) [90]	Educative practice and maternal stress of premature infant: randomized clinical trial	JBI Quasi-experimental	6/9	I
37	Yu (2022) [91]	Sensitivity Training for Mothers with Premature Infants: A Randomized Controlled Trial	JBI RCT	11	I
38	Zhang (2018) [93]	Involvement of Parents in the Care of Preterm Infants: a Pilot Study Evaluating a Family-Centered Care Intervention in a Chinese Neonatal ICU	JBI Quasi-experimental	7/9	I

*References available in manuscript
