# Supplementary material for: Key Components of Parenting Education Interventions for Preterm Infant–Parent Dyads Admitted to the NICU: A Systematic Review
Source: Children (Basel). 2026 Feb 18;13(2):280. doi: 10.3390/children13020280 (PMC12940051; doi:10.3390/children13020280)
Supplement: Supplementary file 1 [file children-13-00280-s001.zip › SUPPL Components within Education Categories.pdf]

Components within Education Content Categories

			NICU environment			Parental aspects								Infant health		Infant behaviour	Infant care										Discharge planning							
Authors	Setting / Country	Effectiveness	NICU environment	NICU staff	NICU: Infection control	Parenting: Father's role	Parenting: Mother emotional changes	Parenting: Mother physical changes	Parenting: Family roles	Parental well-being	Parents: emotional changes	Parenting: interaction	Parenting: attachment	Infant health	Infant health: pain	Infant behaviour	Infant care: Non-specific	Infant care: Positioning	Infant care: diapers	Infant care: bath & clothing	Infant care: cord & eyes	Infant care: sleep	Infant care: temperature	Infant care: massage	Infant care: cuddling	Infant care: feeding	Infant growth & development	Infant growth & development	Discharge preparation	At home: Follow up	At home: Stimulation	At home: Medication	At home: Vaccination	At home: Resuscitation
Bostanabad (2017) [68]	Iran	Yes	X	X		X	X	X						X		X																		
Bracht (2013) [38]	Canada	Yes	X		X					X	X	X	X	X			X								X	X		X	X					
Broom (2017) [50]	Australia	Yes												X											X	X	X		X					X
Chen (2016) [69]	China	Yes							X		X					X													X	X				
Chen (2019) [70]	China, Taiwan	Yes	X			X				X		X		X		X			X	X						X	X	X						X

Cheng (2018) [71]	China, Taiwan	Yes																	X			X	X	X	X	X		X		X			X	X
Evans (2017) [72]	Australia	No			X							X													X	X		X						X
Fotiou (2016) [73]	Greece	Partial				X				X	X	X										X						X						
Hadian (2022) [74]	Iran	Yes	X							X						X										X			X					
Heo (2019) [75]	South Korea	Yes		X		X	X	X						X				X	X						X									
Jafarzadeh (2019) [76]	Iran (Isfahan)	Yes	X									X		X		X		X		X		X			X	X								
Kadiroğlu (2022) [77]	Iran (Eastern Anatolia)	Yes	X	X	X					X				X		X									X	X								
Khanjari (2021) [78]	Iran	Yes					X			X		X	X			X	X			X			X			X			X	X			X	
Lv (2019) [2]	China	Yes												X					X	X	X	X			X	X			X	X				

Maria (2021) [3]	India	Yes			X									X		X			X	X			X		X					X				
Mianaei (2014) [4]	Iran	Yes	X		X									X		X		X		X	X				X	X	X		X	X			X	
Milgrom (2013) [10]	Australia	Yes	X											X		X	X					X						X	X					
Moreno-Sanz (2021) [79]	Spain	Yes														X								X	X									
Morey (2012) [80]	USA	Yes	X		X		X					X		X	X	X		X	X	X			X		X	X						X		
Moudi (2019) [81]	Iran	Yes		X	X				X					X											X	X	X		X					
Mousavi (2021) [82]	Iran (Tehran)	Yes	X		X									X				X	X	X				X	X	X			X	X				
Nieves (2021) [83]	USA	Yes	X	X			X							X		X								X		X								
Ong (2019) [84]	Malaysia	Yes					X			X		X		X		X													X					

Petteys (2018) [85]	USA	Yes	X							X		X		X		X										X	X							
Peyrovi (2016) [86]	Iran	Yes														X											X							
Phianching (2020) [87]	Thailand	Yes	X									X		X		X			X	X	X	X	X			X			X		X	X	X	
Rostami (2020) [88]	Iran	Yes										X		X		X									X	X								
Sivanandan (2021) [89]	India	0	X												X			X				X	X		X	X			X					
Steinhardt (2015) [5]	Germany	Yes										X		X		X						X				X								
Viera (2016) [90]	Brazil	0	X		X														X							X			X					
Yu (2022) [91]	China	Yes				X						X		X						X						X		X		X	X		X	
Yurtsever (2022) [92]	Turkey	0	X														X		X	X			X			X						X		

Zhang (2018) [93]	China	Yes			X									X			X			X				X		X								X
			16	5	10	5	6	2	2	7	4	13	2	22	2	19	5	6	10	12	3	8	7	5	16	24	6	7	14	8	2	3	5	5
			46%	14%	29%	14%	17%	6%	6%	20%	11%	37%	6%	63%	6%	54%	14%	17%	29%	34%	9%	23%	20%	14%	46%	69%	17%	20%	40%	23%	6%	9%	14%	14%
